# Differential Virulence of Aggregatibacter actinomycetemcomitans Serotypes Explained by Exoproteome Heterogeneity

**DOI:** 10.1128/spectrum.03298-22

**Published:** 2022-12-21

**Authors:** Yanyan Fu, Sandra Maaβ, Francis M. Cavallo, Anne de Jong, Erwin Raangs, Johanna Westra, Girbe Buist, Dörte Becher, Jan Maarten van Dijl

**Affiliations:** a University of Groningen, University Medical Center Groningen, Department of Medical Microbiology, Groningen, the Netherlands; b University of Greifswald, Institute of Microbiology, Department of Microbial Proteomics, Greifswald, Germany; c University of Groningen, Groningen Biomolecular Sciences and Biotechnology Institute, Department of Molecular Genetics, Groningen, the Netherlands; d University of Groningen, University Medical Center Groningen, Department of Rheumatology and Clinical Immunology, Groningen, the Netherlands; Centre National de la Recherche Scientifique, Aix-Marseille Université

**Keywords:** *Aggregatibacter actinomycetemcomitans*, exoproteome, serotype, virulence, infection, virulence factors

## Abstract

Aggregatibacter actinomycetemcomitans (*Aa*) is a Gram-negative bacterial pathogen associated with periodontitis and nonoral diseases like rheumatoid arthritis and Alzheimer´s disease. *Aa* isolates with the serotypes a, b, and c are globally most prevalent. Importantly, isolates displaying these serotypes have different clinical presentations. While serotype b isolates are predominant in severe periodontitis, serotypes a and c are generally encountered in mild periodontitis or healthy individuals. It is currently unknown how these differences are reflected in the overall secretion of virulence factors. Therefore, this study was aimed at a comparative analysis of exoproteomes from different clinical *Aa* isolates with serotypes a, b, or c by mass spectrometry, and a subsequent correlation of the recorded exoproteome profiles with virulence. Overall, we identified 425 extracellular proteins. Significant differences in the exoproteome composition of isolates with different serotypes were observed in terms of protein identification and abundance. In particular, serotype a isolates presented more extracellular proteins than serotype b or c isolates. These differences are mirrored in their virulence in infection models based on human salivary gland epithelial cells and neutrophils. Remarkably, serotype a isolates displayed stronger adhesive capabilities and induced more lysis of epithelial cells and neutrophils than serotype b or c isolates. Conversely, serotype c isolates showed relatively low leukotoxicity, while provoking NETosis to similar extents as serotype a and b isolates. Altogether, we conclude that the differential virulence presentation by *Aa* isolates with the dominant serotypes a, b, or c can be explained by their exoproteome heterogeneity.

**IMPORTANCE** Periodontitis is an inflammatory disease that causes progressive destruction of alveolar bone and supporting tissues around the teeth, ultimately resulting in tooth loss. The bacterium Aggregatibacter actinomycetemcomitans (*Aa*) is a prevalent causative agent of periodontitis, but this oral pathogen is also associated with serious extraoral diseases like rheumatoid arthritis and Alzheimer’s disease. Clinical *Aa* isolates are usually distinguished by serotyping, because of known serotype-specific differences in virulence. *Aa* with serotype b is associated with aggressive forms of periodontitis, while isolates with serotypes a or c are usually encountered in cases of mild periodontitis or healthy individuals. The molecular basis for these differences in virulence was so far unknown. In the present study, we pinpoint serotype-specific differences in virulence factor production by clinical *Aa* isolates. We consider these findings important, because they provide new leads for future preventive or therapeutic approaches to fight periodontitis and associated morbidities.

## INTRODUCTION

Aggregatibacter actinomycetemcomitans (*Aa*) is a facultative anaerobic Gram-negative pathogen associated with periodontitis (1). In addition, *Aa* has been associated with extraoral diseases, such as rheumatoid arthritis (2) and Alzheimer´s disease (3). Interestingly, clinical isolates of *Aa* have diverse phenotypes, as underscored by the existence of seven different serotypes (4, 5). These serotypic differences are based on the immunodominant antigenicity of the O-polysaccharide (O-PS) component of the lipopolysaccharide (LPS) in the outer membrane (4, 5). The different *Aa* serotypes encountered in patients with periodontitis can be associated with different geographical locations (6). For instance, serotype a is most identified in the UK and Turkey (7, 8), serotype b in Morocco and India (9, 10), and serotype c in Finland (11). Furthermore, the *Aa* serotypes a, b, and c are globally most dominant, while the other four serotypes are less frequently observed. Importantly, the *Aa* serotypes are associated with periodontal status. While the serotype b is predominant in severe periodontitis, including chronic and aggressive forms of periodontitis, the serotypes a and c are usually encountered in cases of mild periodontitis or healthy individuals (10, 12, 13).

Serotype b isolates were shown to display the strongest immunogenicity *in vitro* compared to the other serotypes. Furthermore, in a murine *Aa* infection model, the serotype b induced higher alveolar bone resorption, and more Th1 and Th17 lymphocytes were detected than when the mice were infected with other *Aa* serotypes (14). Also, inflammatory cytokines and chemokines were produced to higher levels upon infection with *Aa* of serotype b (14). When dendritic cells were stimulated with *Aa*, the serotype b showed the strongest capacity to activate Toll-like receptors 2 and 4, and to trigger cytokine release (15, 16). Likewise, *Aa* with the serotype b displayed a higher capacity to activate T lymphocytes and to induce higher levels of cytokine and chemokine release, which could lead to bone resorption and osteoclast activation (17–20). Lastly, when gingival epithelial cells were challenged with different serotypes of *Aa*, the serotype b induced a higher inhibition of cell growth and higher levels of IL-8 production (21).

The differential virulence of *Aa* with different serotypes as observed *in vivo* and *in vitro* may correlate with the production of particular virulence factors. For instance, higher levels of the leukotoxin A (LtxA) apparently promote the progression of periodontitis (22). In addition, serotype b strains of *Aa*, especially those with the so-called JP2 genotype and *cagE*-positive isolates, can express more LtxA, which has been associated with enhanced virulence (23, 24). In addition to LtxA, other virulence factors have been invoked in host infection and the evasion of host immune defenses (25, 26). Most of these virulence factors are secreted into the extracellular milieu of the bacteria and, therefore, the bacterial “exoproteome” is considered the main reservoir of virulence factors (27).

Although the serotype of *Aa* isolates is closely linked to their clinical presentation, very little is known about possible variations in the overall secretion of virulence factors by this bacterium and possible serotype-specific virulence factor profiles. Such profiles can be distinguished by quantitative proteome analyses, which may help to distinguish highly virulent and less virulent or nonvirulent bacteria. In a previous study, we were able to differentiate *Aa* isolates and derivative strains with serotype b that showed either rough (R) or smooth (S) colony phenotypes by proteomics. Importantly, upon isolation from patients, *Aa* exclusively shows the R phenotype, whereas the S phenotype emerges spontaneously upon continued *in vitro* culturing due to mutations that affect the expression of fimbriae (26, 28). It thus seems that proteomics studies on the overall secretion of virulence factors by R isolates of *Aa* may reflect the clinical presentation of *Aa* more realistically.

**FIG 1:**
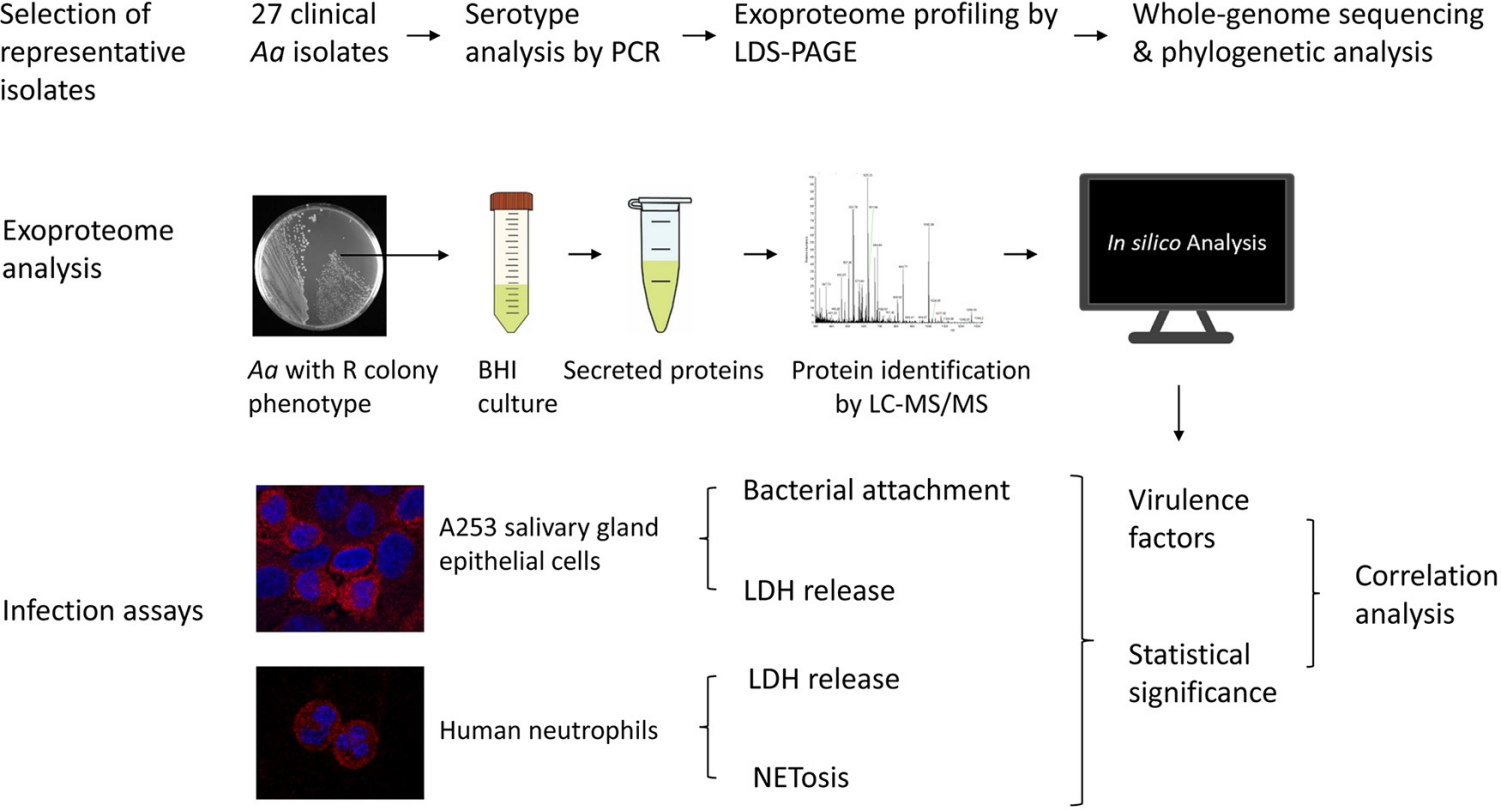
Diagram of the study design. First, representative *Aa* isolates with serotypes a, b, or c were selected from a collection of 27 clinical isolates by PCR-based serotype identification and exoproteome profiling by LDS-PAGE. Genomes of 15 selected *Aa* isolates with serotypes a, b, or c were sequenced and their phylogenetic relationship was determined. Second, bacteria were transferred from a plate to BHI broth and, upon anaerobic standing culture, the secreted proteins were collected by centrifugation and analyzed by LC-MS/MS. Third, the virulence of *Aa* isolates was assayed with A253 salivary gland epithelial cells and human neutrophils by determining attachment to epithelial cells, LDH release as a measure for cytotoxicity, and NETosis. Lastly, the production of virulence factors determined by MS was correlated with bacterial virulence in the different infection assays.

Intriguingly, despite the clear differences in the clinical presentation of *Aa* with different serotypes, it was so far unknown whether or how these differences are mirrored in the overall secretion of virulence factors. Therefore, the present study was aimed at a comparative analysis of the exoproteomes of different R isolates from the three major *Aa* serotypes a, b, and c by liquid chromatography coupled to mass spectrometry (LC-MS/MS), and a correlation of the observed exoproteome profiles with virulence, as outlined in Fig. 1. In brief, our results show indeed significant differences in the exoproteomes of isolates belonging to these three serotypes, which prompted us to also compare their virulence using different infection models. Particularly, infection of A253 salivary gland epithelial cells was used to compare the adhesive and cytotoxic activities of the different *Aa* isolates. In addition, human neutrophils were employed to assay the leukotoxic activity of these isolates. Altogether, our present observations demonstrate that the exoproteome profiles of serotype a, b, or c isolates are very different. Furthermore, the clinical *Aa* isolates of serotype a display stronger adhesive capabilities and induce more lysis of human epithelial cells and neutrophils.

## RESULTS

### Selection of *Aa* serotype a, b, and c study isolates.

**FIG 2:**
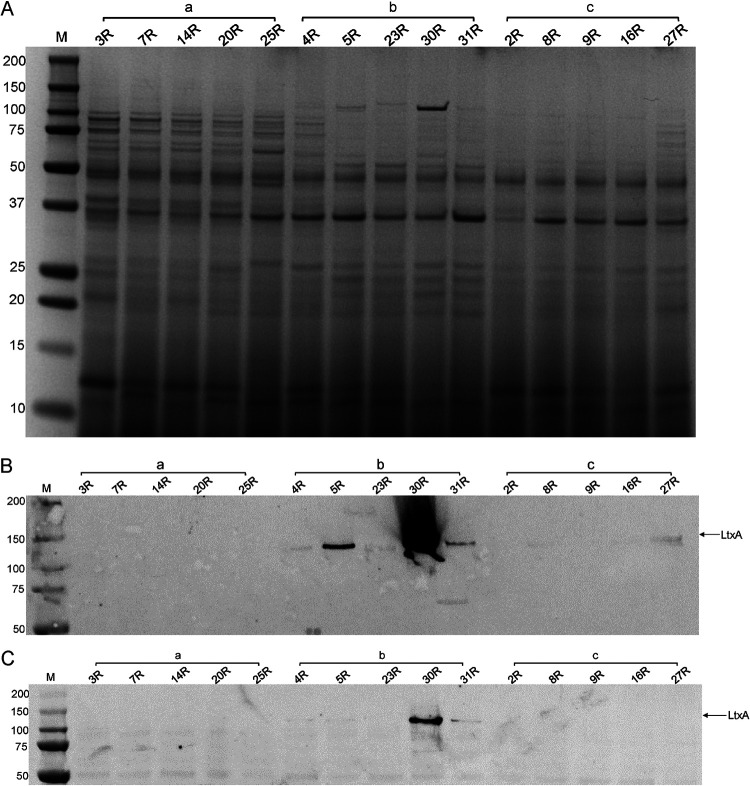
Protein profiling of *Aa* isolates with serotypes a, b, or c. Different *Aa* isolates of serotype a, b, or c were cultured in BHI broth, and extracellular proteins were separated from the bacteria by centrifugation. Extracellular proteins were collected from the growth medium by precipitation with TCA and cellular proteins of the pelleted bacteria were extracted by bead beating. Subsequently, equal amounts of the extracellular proteins were separated by LDS-PAGE and visualized by SimplyBlue-staining (A). The presence of LtxA among the extracellular (B) and cellular proteins (C) was analyzed by Western blotting and immunodetection with a specific monoclonal antibody. LtxA-specific bands are indicated with an arrow. The serotypes of the different analyzed isolates are indicated with a, b, or c. Molecular weights of marker proteins are indicated on the left. Please note that all investigated serotype b isolates, including the isolate 30R that produces large amounts of LtxA, have the non-JP2 genotype.

A major aim of the present study was to compare the exoproteomes and virulence of serotype a, b, and c isolates of *Aa*. To this end, we screened a previously established collection of 27 clinical *Aa* isolates from which we had recently identified seven serotype b isolates (26). Further screening of the strain collection by serotype-specific PCR resulted in the identification of six *Aa* isolates with serotype a, 11 isolates with serotype c, and three isolates with serotype f (see Fig. S1 in the supplemental material). The latter three isolates were excluded from our present analyses. Next, to prescreen the identified isolates with serotypes a and c, we profiled their exoproteomes by LDS-PAGE. As shown in Fig. S1, *Aa* isolates with serotypes a or c showed distinct exoproteome banding patterns upon SimplyBlue gel staining. Based on the differential banding patterns, five serotype a and five serotype c isolates were selected for further analyses, and their exoproteome profiles were compared to those of five recently characterized serotype b isolates (Fig. 2).

Interestingly, the LDS-PAGE analyses showed that the serotype a isolates produce more extracellular proteins compared to most isolates with serotypes b or c. Among the investigated serotype b and c isolates, the isolates 4R and 27R produced the largest number of extracellular proteins. Importantly, additional differences were detectable between isolates with the same serotype. For instance, isolate 30R produced a higher number of extracellular proteins in the 120-kDa range than other serotype b isolates.

Since LtxA is regarded as one of the key virulence factors of *Aa* (29), we assessed the production of LtxA by Western blotting with a monoclonal anti-LtxA antibody (30). As shown in Fig. 2B, LtxA was identified most prominently among the extracellular proteins of serotype b isolates, with isolate 30R secreting particularly large amounts of LtxA. Conversely, LtxA was barely detectable by Western blotting in isolates with the serotypes a or c. Of note, although LtxA-specific bands were not detectable by Western blotting for most of the investigated *Aa* isolates, all isolates did secrete LtxA, as was subsequently shown by MS (Table S1). Furthermore, bacteria-associated LtxA was detectable only for a few of the investigated isolates, with the highest levels being detected in the serotype b isolates 30R and 31R (Fig. 2C). Remarkably, no bacteria-associated LtxA was detected for the serotype b isolate 5R, although it produced more LtxA than the 31R isolate. None of the serotype a or c isolates contained detectable amounts of bacteria-associated LtxA. However, in this context, one should bear in mind that previous studies have shown that the balance between bacteria-associated and secreted LtxA is related to culture conditions (31).

### Genetic characterization of the 15 *Aa* study isolates.

**FIG 3:**
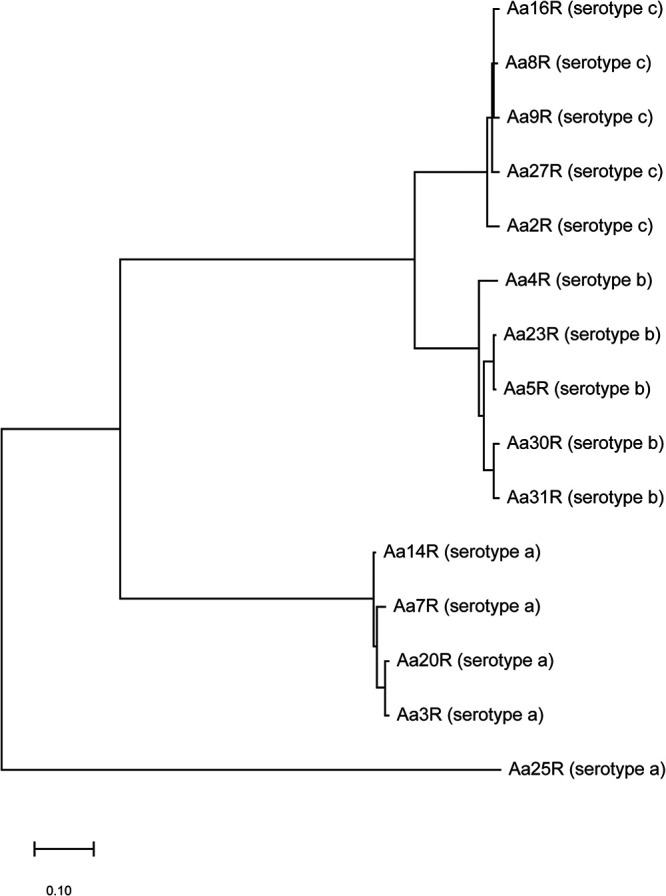
Phylogenetic tree based on the core genomes of 15 isolates of *Aa* according to the maximum likelihood method.

The genomes of the 15 selected *Aa* study isolates were sequenced using the Illumina Miseq and Nanopore MinION platforms. Subsequently, the respective sequence reads were used for hybrid assembly of the genome sequence of each individual isolate. To analyze the evolutionary distance between the isolates with different serotypes, a phylogenetic tree was built, based on the core genomes of the 15 *Aa* isolates (Fig. 3). This analysis revealed that, overall, the serotype a, b, and c isolates represent phylogenetically distinct groups. However, the serotype b and c isolates are more closely related to each other, while the serotype a isolates are more distantly related to the serotype b and c isolates. This is in line with the results from previous pangenome analyses of *Aa* (32–34). Notably, as shown in the phylogenetic tree, the serotype a isolate 25R seems to belong to a separate group.

### Transmission electron microscopy.

**FIG 4:**
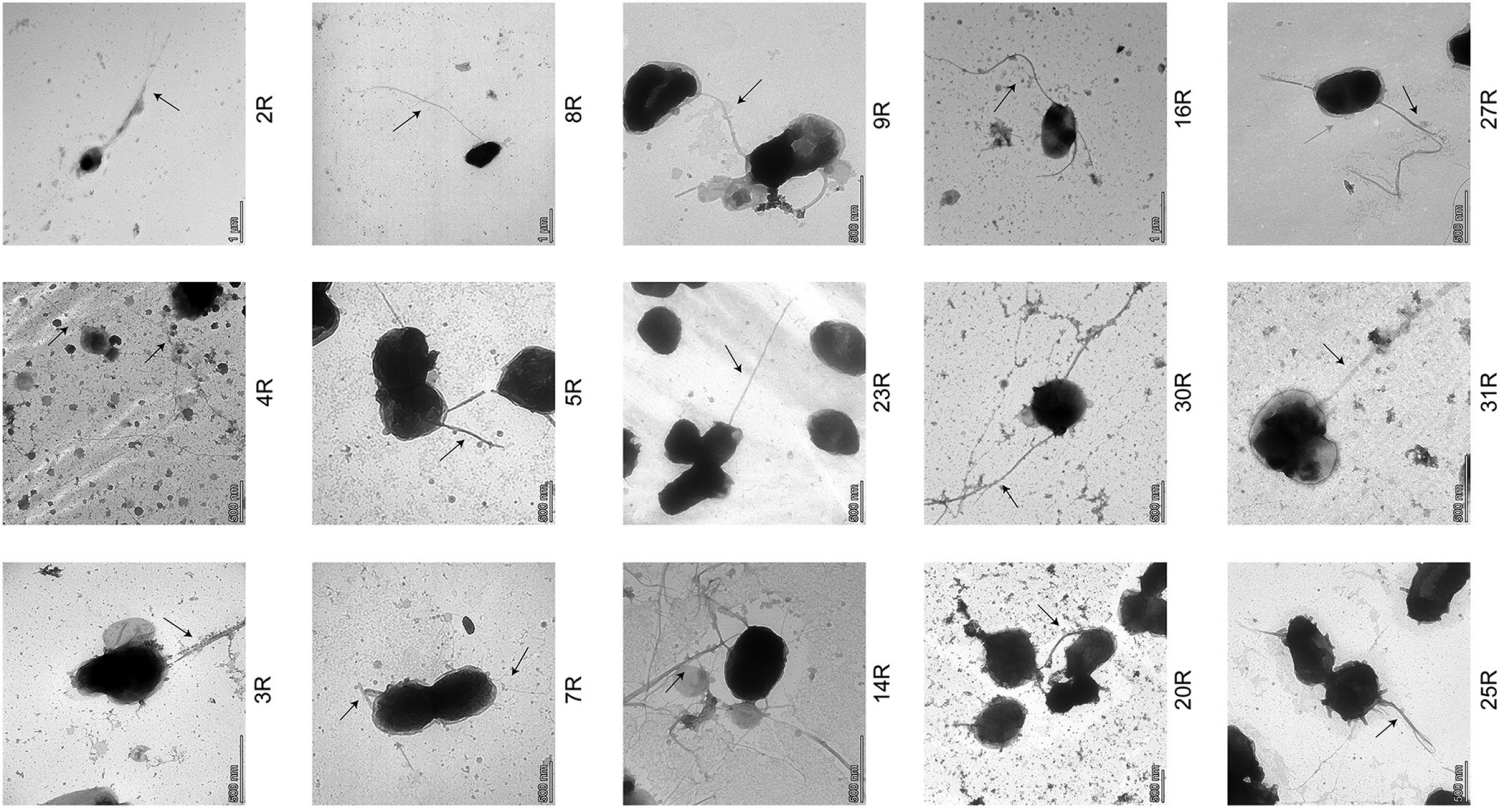
Visualization of *Aa* fimbriation by TEM. Different R isolates of *Aa* were grown on BHI agar for 3 days. Subsequently, the bacteria were collected and washed with PBS, after which the presence of fimbriae was visualized by TEM with negative staining. Scale bars are indicated in each image.

Since the presence of fimbriae on the bacterial cell surface is typical for *Aa* isolates with the R phenotype, we verified the presence of fimbriae for the investigated *Aa* isolates with serotypes a, b, and c by transmission electron microscopy (TEM) with negative staining (Fig. 4). As expected, all investigated *Aa* isolates presented fimbriae on their surface. However, the fimbriae of some isolates had different morphologies. For instance, some isolates showed fimbriae with a web-like structure, as exemplified by isolate 14R. In contrast, long and thin fimbriae were detected for isolates 8R and 23R, whereas bundles of fimbriae were detected for isolates 4R, 5R, and 25R. Furthermore, some *Aa* isolates, such as 7R and 27R, displayed short extensions associated with the outer membrane.

### Serotype a isolates of *Aa* show higher exoproteome heterogeneity than serotype b or c isolates.

To investigate the heterogeneity of extracellular proteins produced by different *Aa* isolates with different serotypes, the exoproteomes of the five study isolates of each serotype were analyzed by MS. To this end, the extracellular proteins of each isolate were collected upon 40 h of growth in brain heart infusion (BHI) broth. Subsequently, their identity and quantity were determined by LC-MS/MS. Furthermore, the predicted subcellular localization of each identified protein was determined to understand through which pathways it had most likely reached the extracellular milieu of the bacteria. The identified proteins, their respective normalized abundance values and the predicted localization are listed in Table S1. A total of 425 extracellular proteins were identified for all investigated *Aa* isolates. Overall, 309 proteins (72.7%) were predicted to have a cytoplasmic localization, indicating that they belong to the class of extracellular cytoplasmic proteins (ECPs). Additionally, 79 proteins (18.6%) were predicted to have a periplasmic localization, 31 proteins (7.2%) were predicted to have an outer membrane localization, three proteins were predicted to be localized to the inner membrane, and three proteins were predicted to be secreted.

**FIG 5:**
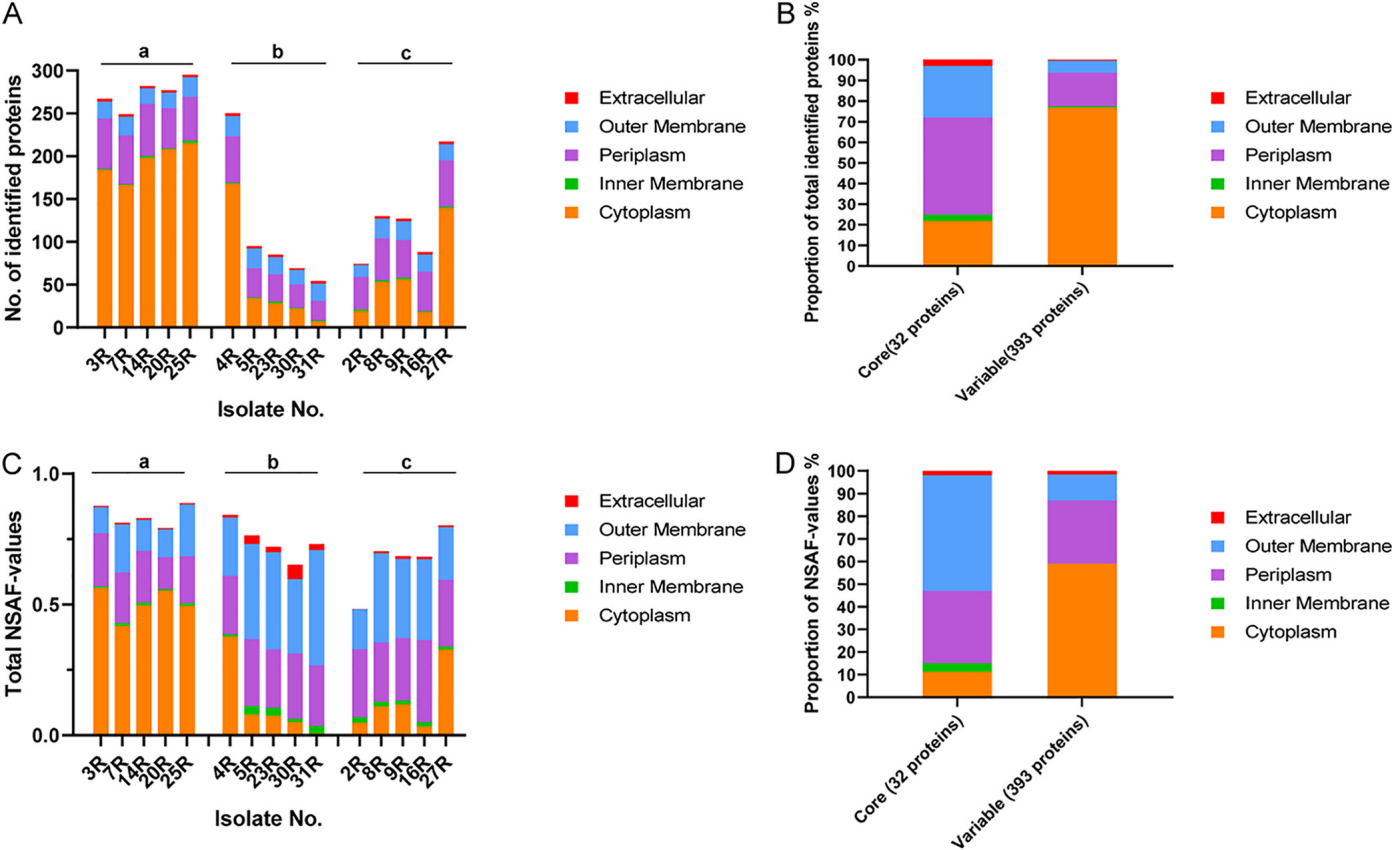
The predicted subcellular localization of all identified extracellular proteins. The subcellular localization of all extracellular proteins identified in the growth medium fractions was predicted with different bioinformatic tools and manual curation based on literature data. (A) Numbers of identified proteins with a particular predicted localization per isolate. (B) Proportion of predicted protein localization based on the numbers of identified proteins in the core and variable exoproteomes of the investigated isolates. (C) Relative amounts of proteins of each isolate with a particular predicted localization based on NSAF values. (D) Proportion of predicted protein localizations based on the NASF values for the core and variable exoproteome.

Interestingly, the numbers of identified proteins were found to differ greatly between serotypes a, b, and c (Fig. 5A). The largest numbers of extracellular proteins were identified for serotype a isolates with a maximum number of 295 different extracellular proteins being identified for isolate 25R. This number is significantly larger than the 179 extracellular proteins previously reported for the rough serotype a strain D7S (35). In contrast, the lowest numbers of extracellular proteins were identified for the serotype b isolates with a minimum of 54 different extracellular proteins being identified for isolate 31R. However, the serotype b isolate 4R formed an exception with 250 identified extracellular proteins, similar to isolates with the serotype a. These numbers are close to identical to the numbers of extracellular proteins of these serotype b strains that we previously reported (26). The numbers of extracellular proteins identified for serotype c isolates ranged between those identified for the serotype a and b isolates, with 74 extracellular proteins identified for isolate 2R and 217 proteins identified for isolate 27R. Notably, the numbers of identified ECPs showed the greatest variation between the different isolates, whereas the numbers of identified extracellular proteins with other predicted subcellular localizations were more comparable between different isolates. For instance, the numbers of ECPs of serotype a isolates ranged from 215 (25R) to 166 (7R), while the ECPs of serotype b isolates ranged from 168 (4R) to 7 (31R), and the ECPs of serotype c isolates from 139 (27R) to 18 (16R).

To further define the exoproteome differences for *Aa* isolates with different serotypes, the amounts of proteins with different predicted extracellular localization were also assessed based on the respective NSAF-values as determined by MS. As shown in Fig. 5C, the genuine secreted proteins and the proteins with a predicted outer membrane localization of serotype b isolates were found to be more abundant than the respective proteins from serotype a and serotype c isolates. Interestingly, judged by the NSAF values, the identified ECPs are much less abundant in terms of their amounts than suggested by the numbers of ECP identifications. Conversely, the abundance of outer membrane proteins, as judged by the NSAF values, is much higher than suggested by the numbers of outer membrane protein identifications; for some isolates, the outer membrane proteins even outnumbered the ECPs in terms of cumulative NSAF values (Fig. 5C).

Among the total number of 425 identified extracellular *Aa* proteins, only 32 extracellular proteins were detected for all investigated isolates. We regard these 32 proteins as the core exoproteome of *Aa*. As shown in Fig. 5B, predicted periplasmic proteins account for the highest proportion (46.9%) of the extracellular core proteins, followed by ECPs (21.9%). Only one extracellular core protein has a predicted inner membrane localization (i.e., TadG), and another one is predicted to be secreted (i.e., LtxA). However, it should be mentioned here that LtxA was also reported to be localized to the bacterial outer membrane and so-called outer membrane vesicles (31). Furthermore, 35 extracellular proteins were identified in at least 80% of the investigated *Aa* isolates. Altogether, 393 identified extracellular proteins belong to the variable *Aa* exoproteome. ECPs account for the highest proportion (76.8%) of variable exoproteins, followed by periplasmic proteins (16.3%). Merely two extracellular proteins were predicted to be located on the inner membrane (TorC, TadA), and two were predicted to be actively secreted (CdtA, CdtB).

To appreciate the identified core and variable extracellular proteins in terms of protein abundance, their cumulative NSAF values were determined by MS (Fig. 5D). Unlike the numbers of protein identifications, the identified outer membrane proteins had the highest relative abundance among the core extracellular proteins. Among the variable extracellular proteins, the ECPs displayed the highest abundance, albeit that their proportion was relatively lower than the numbers of ECPs among the variable extracellular proteins (Fig. 5B and D). Conversely, the relative proportion of periplasmic proteins in terms of abundance was higher among the variable extracellular proteins.

**FIG 6:**
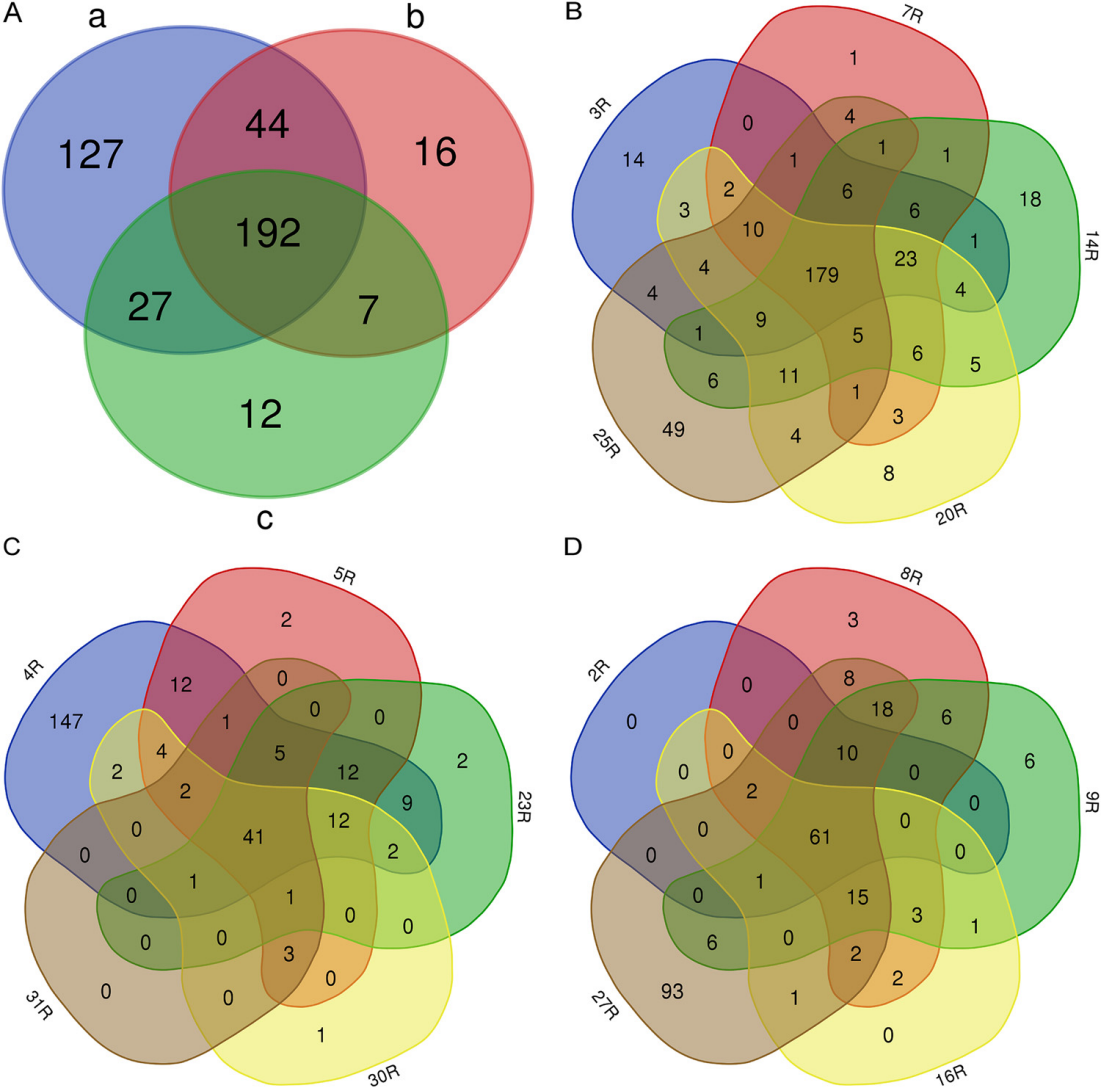
The comparison of the numbers of extracellular proteins detected in *Aa* isolates with serotypes a, b, or c. Venn diagrams display the numbers of common and unique identified proteins for isolates with the three different serotypes. The numbers of identified proteins are compared between the three serotypes (A), or for individual isolates of serotype a (B), serotype b (C), or serotype c (D).

Since the different isolates of *Aa* showed significant differences in the number of identified extracellular proteins, with relatively more extracellular proteins identified for the serotype a isolates, we assessed the numbers of common and unique identified proteins for the three serotypes and for the individual isolates belonging to each serotype. Venn diagrams were used to visualize the similarities and differences. As shown in Fig. 6A, 192 extracellular proteins were common to all isolates of the three serotypes. Furthermore, 127 extracellular proteins were unique for serotype a isolates, of which 113 proteins were ECPs. In contrast, 16 extracellular proteins were unique for serotype b isolates (including 10 ECPs), and 12 were unique for serotype c isolates (including 8 ECPs).

For serotype a isolates, 179 common extracellular proteins were identified (Fig. 6B), which included 123 ECPs and 37 periplasmic proteins. Among the serotype a isolates, the 25R isolate displayed the highest number of unique proteins, namely, 49 of which 41 proteins were ECPs. For serotype b isolates 41 common extracellular proteins were identified (Fig. 6C). In this category, the 4R isolate displayed 147 extracellular proteins, of which 126 were ECPs. Lastly, 61 proteins were common for serotype c isolates (Fig. 6D). Here, the 27R isolate presented 93 extracellular proteins, including 86 ECPs. Altogether, these numbers highlight the fact that most of the unique identified proteins for each isolate were ECPs.

**FIG 7:**
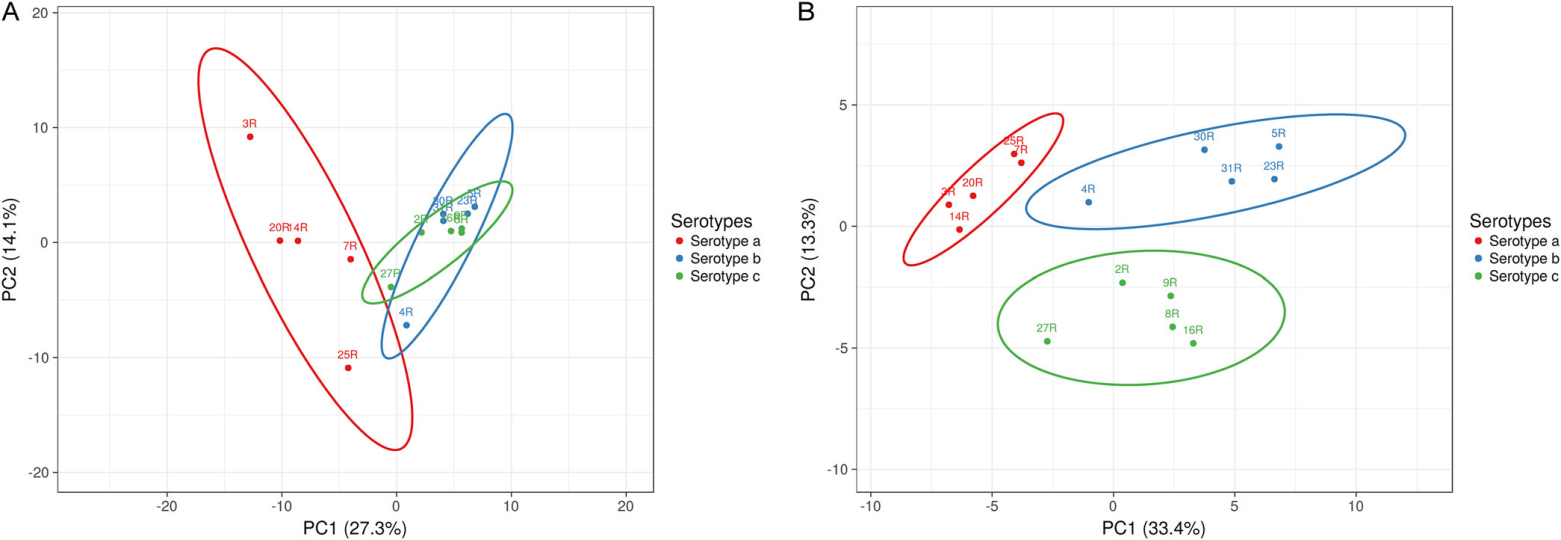
Principal-component analysis (PCA) of all identified extracellular proteins of serotype a, b, and c isolates. The PCA plots are based on the normalized spectral counts of extracellular proteins identified for the *Aa* isolates belonging to the serotypes a, b, and c. (A) All identified extracellular proteins. (B) All identified extracellular proteins except the ECPs.

To investigate the exoproteome heterogeneity among *Aa* isolates with the three different serotypes, a principal-component analysis (PCA) was performed based on the normalized spectral counts (Fig. 7A). The PCA results clearly separate the serotype a isolates from the serotype b and c isolates. On the other hand, the serotype b and c isolates are similarly judged by the PCA. Furthermore, the serotype a isolates are more heterogeneous than isolates from the two other serotypes. Judged by the PCA, the 3R and 25R isolates with serotype a are more distantly related to the other isolates with serotype a. Furthermore, the PCA indicates a more distant relationship between the 4R isolate and the other serotype b isolates, while for the 27R isolate, a more distant relationship with other serotype c isolates is indicated. Since ECPs formed a prominent class among the identified extracellular proteins of the different *Aa* isolates, we also investigated their contribution to the relationships between the isolates belonging to different serotypes. To this end, another PCA was performed on the normalized spectral counts of the identified extracellular proteins, except the ECPs (Fig. 7B). Interestingly, this PCA allowed a clear separation of the serotype a, b, and c isolates. Moreover, in this case, the serotype a isolates showed less heterogeneity than the serotype b or c isolates, with the serotype b isolates showing the highest heterogeneity. Overall, we conclude that all extracellular proteins contribute to the distinctive exoproteome profiles of *Aa* isolates belonging to the three different serotypes, but that ECPs shape the appearance of the relationships between isolates belonging to the different serotypes.

When focusing on the relative abundances of extracellular proteins identified for each *Aa* isolate, we noticed that outer membrane proteins (OMPs) represented a very prominent protein fraction. Therefore, we performed another PCA to assess the influence of OMPs on the clustering of isolates with different serotypes. As shown in Fig. S2, the separation of the serotype a, b, and c isolates was comparable to that of the PCA based on all identified extracellular proteins.

Voronoi treemaps were drawn to visualize the functional categories to which all 425 identified proteins of the investigated *Aa* isolates belong. To this end, their functions were predicted based on gene ontology (GO) terms using InterPro, which allowed a grouping of the identified proteins into four top-level functions and 23 second-level functions (see Fig. S3 in the supplemental material). As shown in Fig. S3, the top five functions are “catalytic activity,” “metabolic activity,” cellular processes,” “binding,” and “cellular anatomical entity.” The most abundant function identified for extracellular proteins of serotype a isolates is related to catalytic activity, while the most abundant function for extracellular proteins from serotype b and c isolates is related to cellular anatomical entity. The common most abundant proteins shared by isolates of the three serotypes are OmpA, the lipoprotein “GNA1870-like protein Lipoprot_C,” the phospholipid-binding protein MlaC, and the outer membrane lipoprotein Pcp.

**FIG 8:**
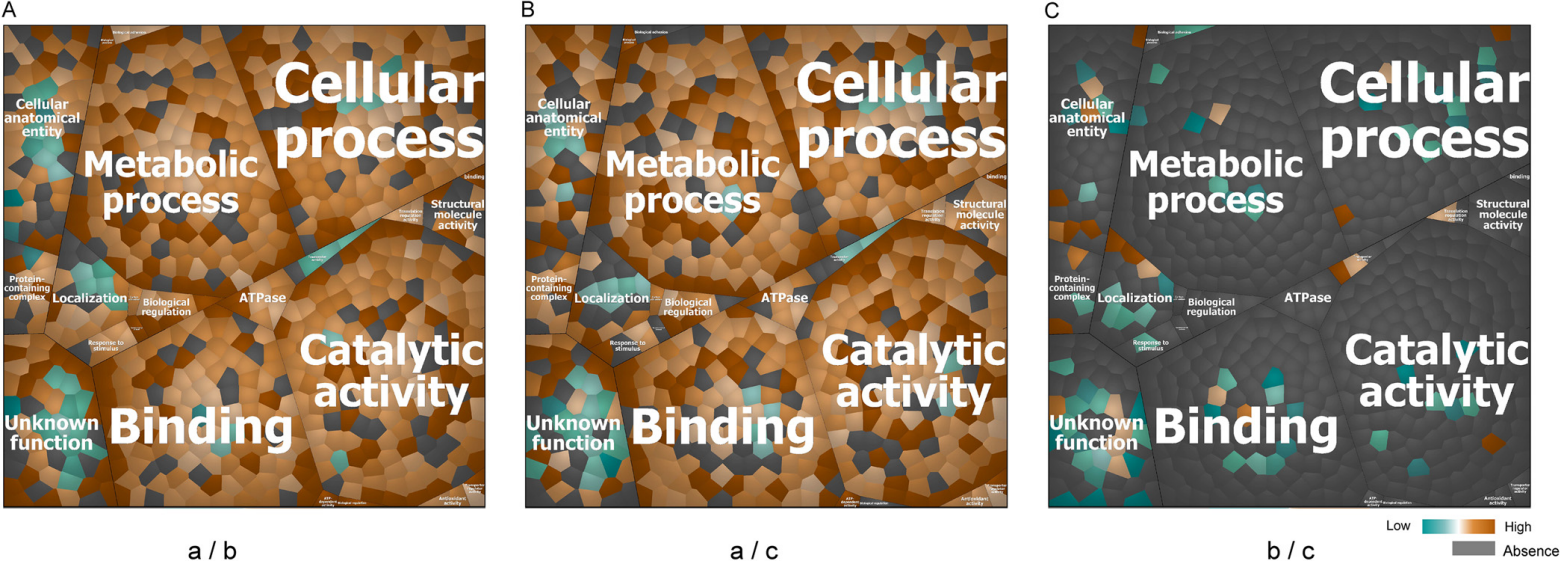
Functional characterization of the extracellular proteins from all investigated *Aa* isolates. The functional categories to which identified extracellular proteins belong were first defined by GO terms and then visualized with Voronoi treemaps based on the “second level functions.” To this end, functionally related gene products were assigned to the same cluster and partitioned into weighted polygons with an area proportional to the relative weight of the respective functional category. To highlight the differences between the identified proteins from isolates belonging to pairs of serotypes, only the differential proteins are displayed in the treemaps. (A) Comparison of the relative abundance of extracellular proteins from serotype a and b isolates. (B) Comparison of the relative abundance of extracellular proteins from serotype a and serotype c isolates. (C) Comparison of the relative abundance of extracellular proteins from serotype b and c isolates. Orange marks the proteins with higher abundance in the first-mentioned serotype, while blue marks the proteins with higher abundance in the later serotype (a/b, a/c, or b/c for A, B, and C, respectively). Absent protein ratios are marked in gray.

To pinpoint the differences between the serotype a, b, and c isolates in terms of functional categories of the extracellular proteins identified at different levels, we performed pairwise comparisons and visualized the results for the second level functional categories using Voronoi treemaps (Fig. 8). The respective protein names are specified in Fig. S3. An interesting observation was that the most pronounced differences in relative protein abundance were observed upon comparison of the proteins from serotype a isolates with those from serotype b or c isolates. In contrast, the relative abundance of identified proteins from serotype b and c isolates was relatively similar. Specifically, 57 identified proteins from serotype a isolates were not detected for serotype b isolates, while only two proteins from the serotype b isolates were not detected for serotype a isolates. Furthermore, compared with serotype b isolates, the extracellular proteins from serotype a isolates with the highest relative abundance were Gmd, GlmS, Tal, Ggt, AhpC, and TyrS. Conversely, compared with serotype a isolates, the extracellular proteins from serotype b isolates with the highest relative abundance were LtxA, LsrB, SC1000_07355, BamA, and Aae. A total of 28 extracellular proteins were more abundant in isolates with serotype b compared to serotype a isolates, and 17 of these proteins were known virulence factors. Fifty-nine extracellular proteins were not detected for serotype c isolates compared with serotype a isolates, while only one protein from serotype c isolates was not detectable in isolates with serotype a. Furthermore, compared with serotype c isolates, the relatively most abundant extracellular proteins from serotype a isolates were FumC, Tig, GltX, TpiA, and SerS. Conversely, the relatively most abundant extracellular proteins from serotype c isolates were TroA_e, FecB, TonB-hemin, DUF5358, and OMPP1. Compared to serotype a isolates, 27 extracellular proteins from the serotype c isolates were more abundant, and 12 of these proteins were known virulence factors. Lastly, in the comparison of extracellular proteins from the serotype b and c isolates, only 43 proteins were considered to have a significantly different abundance, of which 13 proteins were more abundant among serotype b isolates. Four extracellular proteins of serotype b isolates were not detected for serotype c isolates while, conversely, eight proteins of serotype c isolates were not detected for serotype b isolates.

### *Aa* isolates belonging to different serotypes show distinct virulence factor profiles.

**FIG 9:**
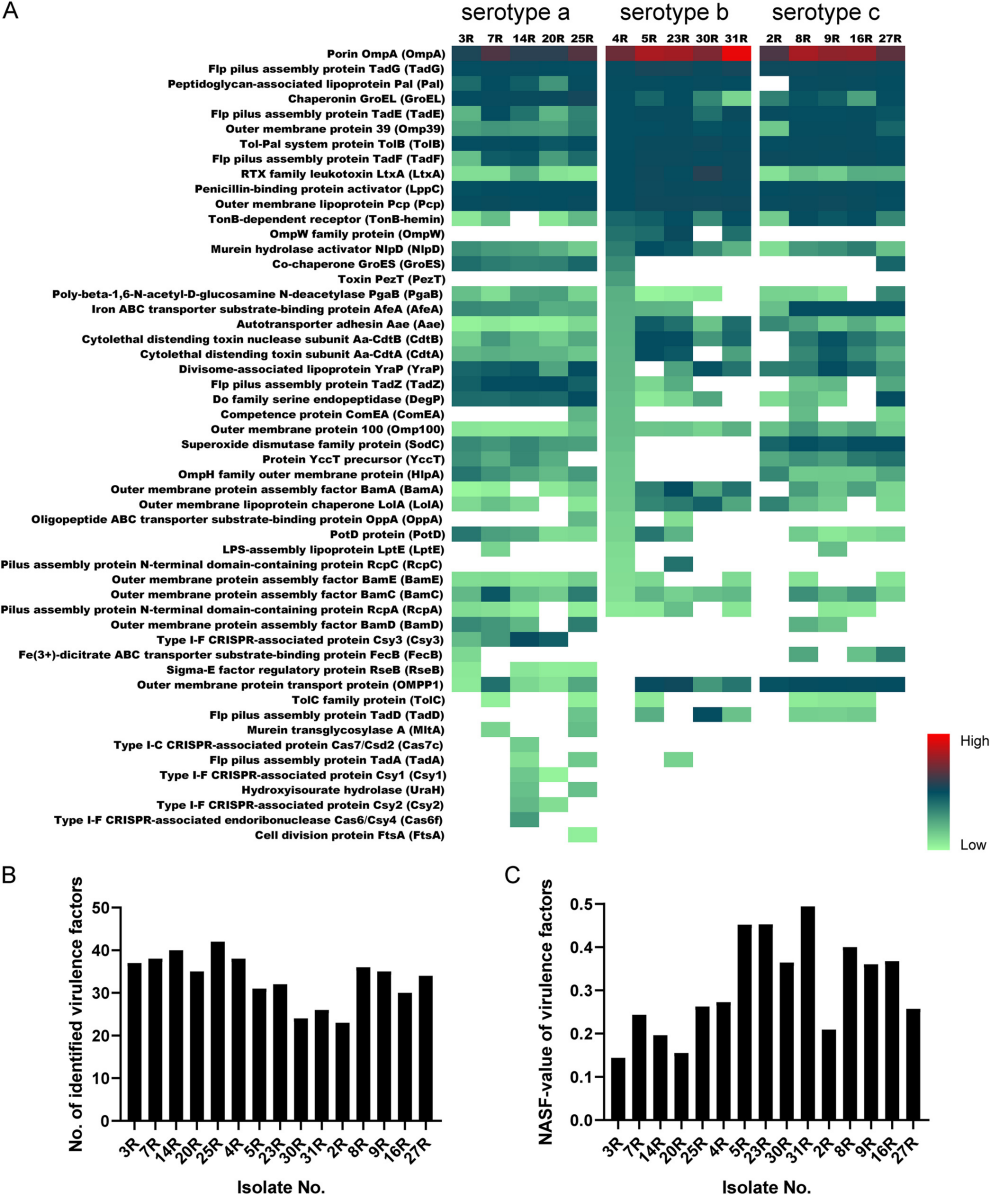
Differential abundance of known virulence factors in the growth media of all investigated *Aa* isolates. (A) Heatmap based on the normalized spectral counts of the respective virulence factors. A total of 53 known virulence factors was identified in all investigated *Aa* isolates with serotypes a, b, or c. (B) Numbers of identified virulence factors per isolate. (C) Cumulative NSAF values of identified virulence factors per isolate.

A heatmap was used to visualize the relative abundance of known virulence factors of *Aa* isolates with the serotypes a, b, or c (Fig. 9A). A total of 53 virulence factors were identified in the growth media of all investigated *Aa* isolates. These virulence factors play roles in bacterial adhesion and invasion, biofilm formation, immune evasion, cytotoxicity, immunoreactivity and proinflammatory activity, or drug targeting (36). One interesting finding is that proteins belonging to particular complexes were recognized in virulence factor profiles, such as Tad proteins that form fimbriae involved in adherence, the BAM complex for protein translocation across the outer membrane, the cytolethal distending toxin (CDT), and the clustered regularly interspaced short palindromic repeats (CRISPR)-associated (Cas) proteins. Thirteen known extracellular virulence factors were identified for all investigated *Aa* isolates, including some well-studied proteins, like OmpA and LtxA. Moreover, OmpA was the most abundant protein identified for each of the investigated *Aa* isolates. However, clear differences in abundance were evident for other core virulence factors. For instance, LtxA was more abundant in the media of serotype b isolates compared to serotype a and c isolates, while GroEL was more abundant in the media of serotype a isolates compared to serotype b or c isolates. Furthermore, OMPP1 was more abundant in the media of serotype c isolates than in media of serotype a or b isolates. Interestingly, more known virulence factors were identified for serotype a isolates, which ranged in numbers from 42 (25R) to 35 (20R) virulence factors. In contrast, the numbers of identified virulence factors of serotype b isolates ranged between 38 (4R) and 24 (30R), whereas these numbers ranged from 35 (9R) to 23 (2R) for serotype c isolates (Fig. 9B). Some virulence factors were exclusively identified in isolates belonging to particular serotypes. For instance, RseB and the CRISPR complex proteins were only identified in the media of serotype a isolates, whereas OmpW was only identified for serotype b isolates. No virulence factor was exclusively identified for serotype c isolates. Lastly, some known virulence factors were only identified for one or two isolates. For instance, MltA was only identified for the 7R and 25R isolates, and FtsA was only identified for the 25R isolate.

To better appreciate the differences in identified virulence factors produced by isolates that belong to the three different serotypes, we also investigated the abundance of these virulence factors based on NASF values (Fig. 9C). Interestingly, judged by the abundance of extracellular virulence factors, serotype b isolates secreted more of the respective proteins than isolates belonging to the other two serotypes, and the amounts of extracellular virulence factors of serotype a isolates were substantially lower than observed for isolates belonging to serotypes b and c.

Since some important known virulence factors of *Aa* were not identified for particular isolates in our exoproteome analyses, we verified whether this could relate to the absence of the respective genes. To this end, we evaluated the respective genome sequences for the presence or absence of the genes encoding these virulence factors. Importantly, genes for most of the apparently absent virulence factors were represented in the genomes of the respective isolates. However, the *ompW* and *cdtA* genes were absent from the 2R isolate. Further, the *pezT* gene was absent from all serotype a and c isolates and the two serotype b isolates 30R and 31R. Lastly, the *csy1*, *csy2*, *csy3*, *cas6f*, and *csas7c* genes for proteins belonging to the CRISPR complex were absent from all serotype b and c isolates as well as the serotype a isolate 25R.

### Virulence properties of *Aa*.

**FIG 10:**
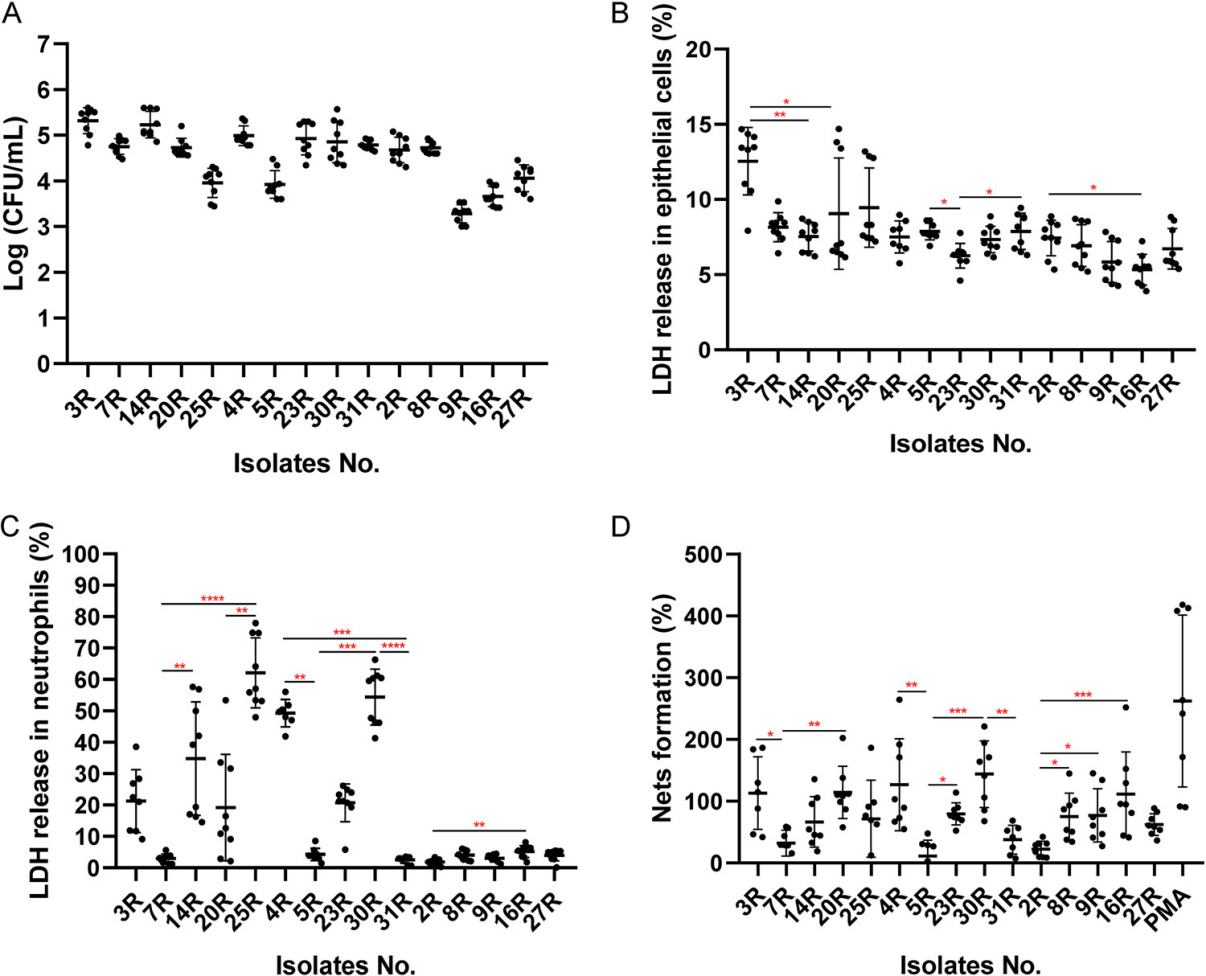
Impact of *Aa* in different infection models. Adhesion of *Aa* to human salivary gland epithelial cells (A). Release of LDH by human epithelial cell (B) or neutrophils (C). NETs formation by human neutrophils (D). The statistical significance of data from CFU measurements was assessed using two-way ANOVA tests and subsequent Šidák multiple-comparison tests (see Table S2 in the supplemental material). The statistical significance of data from LDH release and NETosis assays was assessed by Kruskal-Wallis tests with subsequent Dunn’s or the Dunnett’s multiple-comparison tests (*, *P* < 0.03; **, *P* < 0.002; ***, *P* <0.0002; ****, *P* <0.0001).

Since we observed a substantial heterogeneity in the exoproteomes of *Aa* isolates belonging to the three different serotypes, especially with respect to virulence factors, we decided to evaluate possible differences in the virulence of these isolates. Specifically, we examined their virulence toward human salivary gland A253 epithelial cells and human neutrophils. Of note, to assess the statistical significance of the observed effects, we first compared the differences between serotypes and subsequently the differences observed for individual isolates with serotypes a, b, or c (Table S2). In a first approach, we tested the ability of *Aa* to adhere to *in vitro* cultured A253 epithelial cells. To this end, the cells were infected with *Aa*, and the CFU of cell surface-attached bacteria were subsequently measured. As shown in Fig. 10A, no differences in epithelial cell adhesion were observed between serotypes a and b, while the observed adhesion for serotype a or b isolates was significantly higher than that of serotype c isolates. In particular, the isolates 3R and 14R exhibited a higher ability to adhere to the epithelial cells than the other three *Aa* isolates with serotype a. Furthermore, the serotype a isolates 7R and 20R showed higher adherence than the 25R isolate. Among the serotype b isolates, isolate 5R was less able to adhere to epithelial cells than the other four serotype b isolates. As for serotype c, the 2R and 8R isolates showed higher adherence to epithelial cells than the other three serotype c isolates, with the 27R isolate showing higher adherence than the 9R and 16R isolates, and the 16R isolate showing higher adherence than the 9R isolate. To investigate possible cytotoxic effects of the different *Aa* isolates, epithelial cells were infected with *Aa* for 6 h and the amounts of released lactate dehydrogenase (LDH) were measured (Fig. 10B). Serotype a isolates showed a higher cytotoxicity than serotype b or c isolates, while serotype b isolates were more cytotoxic than serotype c isolates. Among the serotype a isolates, the 3R isolate provoked more LDH release than the 14R or 20R isolates. LDH release caused by the serotype b isolate 23R was lower than the LDH release caused by the two other serotype b isolates 5R or 31R. For serotype c isolates, the highest LDH release was observed for the isolate 2R, whereas the 16R isolate provided the lowest level of LDH release.

For neutrophils, no differences in LDH release were observed upon incubation with serotype a or b isolates, but these isolates provoked overall much more LDH release from the neutrophils than serotype c isolates. Among the serotype a isolates, 7R was less toxic toward neutrophils than the 14R or 25R isolates, while isolate 20R was less toxic than isolate 25R. Furthermore, the serotype b isolates 4R and 30R provoked more neutrophil lysis than the 5R and 31R isolates. Among the serotype c isolates, the 2R isolate was less toxic for neutrophils than the 16R isolate. To further investigate how different *Aa* isolates impacted the neutrophils, the formation of neutrophil extracellular traps (NETs) was measured at 2 h postinfection using a high-throughput NETosis assay. Interestingly, no significant differences were observed between serotype a, b, and c isolates. Nevertheless, NETs formation triggered by the serotype a isolates 3R or 20R was higher than that triggered by isolate 7R. For serotype b isolates, NETs formation by isolate 5R was lower than that triggered by isolates 4R or 30R, while isolate 30R triggered more NETs formation than isolate 31R. Among the serotype c isolates, the 8R, 9R, or 16R isolates provoked higher levels of NETs formation than isolate 2R.

### Correlations between known secreted virulence factors and differential behavior of *Aa* isolates in infection models.

To investigate whether the secretion of particular virulence factors might have a dominant influence on virulence traits of *Aa* in the different infection models, we assessed possible correlations between the detected virulence factors and the adhesion to or killing of epithelial cells, neutrophil killing, and NETosis (Table 1 and Table S3). This showed that the presence of TadA and the “pilus assembly protein N-terminal domain-containing protein” RcpC, as well as the higher abundance of TadG and the autotransporter adhesin Aae were associated with higher degrees of *Aa* adhesion to epithelial cells. Furthermore, the detection and higher abundance of TadD and the outer membrane lipoprotein chaperone LolA were associated with elevated killing rates of epithelial cells. In the neutrophil infection model, the detection or higher abundance of TadA, TadD, and the oligopeptide ABC transporter substrate-binding protein OppA were associated with higher neutrophil lysis as measured by LDH release, whereas the presence or a relatively high production of TadD was associated with increased NETs formation.

**TABLE 1:** Extracellular virulence factors of *Aa* significantly or uniquely associated with virulence in cell infection models

Infection model	Protein name
Adhesion to epithelial cells	Flp pilus assembly protein TadA (TadA)
	Pilus assembly protein N-terminal domain-containing protein RcpC (RcpC)
	Flp pilus assembly protein TadG (TadG)
	Autotransporter adhesin Aae (Aae)
Epithelial cell killing	Flp pilus assembly protein TadD (TadD)
	Outer membrane lipoprotein chaperone LolA (LolA)
Neutrophil killing	Flp pilus assembly protein TadA (TadA)
	Flp pilus assembly protein TadD (TadD)
	Oligopeptide ABC transporter substrate-binding protein OppA (OppA)
NETosis	Flp pilus assembly protein TadD (TadD)

## DISCUSSION

Serotyping is a common and very important approach to distinguish particular bacterial or viral isolates, understand their biological evolution, track outbreaks, and even to monitor the burden of diseases (37–39). Moreover, the serotyping mirrors the immune responses to pathogens, thereby providing clues concerning their interactions with the immune system (40). In the present study, we therefore sought to compare the extracellular protein profiles of clinical *Aa* isolates with different serotypes, and to correlate the identified exoproteome differences to their behavior in distinct infection models. The rationale for focusing our attention in these studies on extracellular proteins was that the exoproteome is a major reservoir for bacterial virulence factors (27, 35).

The dominant serotypes in our collection with clinical *Aa* isolates from Dutch patients with severe periodontitis were the serotypes a, b, and c, which is in accordance with other reports on the prevalence of particular *Aa* serotypes (41). A phylogenetic analysis of the isolates selected for the present study based on their genome sequences showed that each serotype formed a distinct cluster, but isolates with serotypes b and c were more closely related to each other than to isolates with the serotype a that represent a more distinct lineage. Furthermore, the serotype a isolate 25R turned out to be quite distinct from other serotype a isolates. The latter observation is consistent with other previously reported observations, suggesting that isolates of serotype a are more heterogeneous than *Aa* isolates with other serotypes (32, 33, 42).

Consistent with the genomic distinction of *Aa* isolates with serotypes a, b, or c, the exoproteome profiles of these isolates were also significantly different. Especially, the exoproteomes of the serotype a isolates differed from those of the serotype b and c isolates because the serotype a isolates were found to present more extracellular proteins than isolates with serotypes b or c. This distinction is mostly related to higher numbers of ECPs in the growth media of serotype a isolates. Of note, the release of ECPs from the bacterial cells is not exclusively restricted to *Aa*, because another notorious oral pathogen, Porphyromonas gingivalis, also displayed this phenomenon (43). Likewise, different isolates of the Gram-positive bacterial pathogen Staphylococcus aureus, the cell factory for industrial enzymes Bacillus subtilis, or even eukaryotic cells are known to produce many ECPs (44, 45). It is important to note here that different mechanisms have been implicated in the release of ECPs into the extracellular milieu, including cell death, autolysis, membrane weakening by toxins, and cell lysis due to the induction of prophages (45–47). For S. Aureus, it was also reported that upregulation of the cell division protein FtsA promoted the release of the cytoplasmic proteins into the growth medium, and it was proposed that the excretion of cytoplasmic proteins coincided with the moment of cell division when the bacterial cell integrity may be partially compromised (48). In our present study, we detected the FtsA protein only in the medium of the 25R isolate, which makes it difficult to correlate FtsA to the detection of ECPs. In a recent study, we observed that the derivative S strains of *Aa*, which displayed a smooth colony phenotype due to reduced fimbriation, also released more ECPs than the corresponding clinical R isolates with the rough colony phenotype (26). Although all investigated isolates in the present study have the rough colony phenotype, it is thus conceivable that differences in the fimbriation of the serotype a isolates compared to the serotype b and c isolates are responsible for the observed differences in ECP abundance, although this is not directly evident from our TEM analyses. On the other hand, relatively smaller amounts of the fimbrial TadD, TadE, and TadF proteins were detected in the growth media of the serotype a isolates, which would be consistent with the notion that a relatively lower degree of fimbriation is associated with a relatively higher abundance of ECPs (26). Another distinguishing feature of the serotype a isolates is that their growth media contained elevated levels of the Do family serine endopeptidase DegP and the YccT protein. Both DegP and YccT are required for appropriate responses to environmental stresses in many different bacterial species (49, 50). In particular, DegP is involved in periplasmic protein quality control, aiding in the refolding or degradation of unfolded proteins (51). YccT was shown to play an important role in management of the cellular responses to peroxide stress in Salmonella enterica (50). The presently observed relatively high levels of DegP and YccT in the serotype a isolates of *Aa* thus suggest that these isolates have adapted to particular oxidative stresses that could damage extracytoplasmic proteins and affect the integrity of the bacterial cell envelope. This may explain an elevated release of ECPs into the extracellular milieu of the bacteria. In this context, it is noteworthy that, irrespective of their precise mechanism of release from the bacteria, several ECPs were shown to serve both intracellular and extracellular “moonlighting” functions. This includes proteins with a role in the bacterial metabolism, like enolase and aldolase (Table S1), that also have a role in the bacterial adhesion to host cells and tissues (52, 53).

Outer membrane proteins (OMPs) are an important class of virulence factors in Gram-negative bacteria. The OMPs of *Aa* have multiple functions, including adhesion and invasion of host cells, serum resistance and immune evasion (54, 55). Moreover, OMPs of *Aa* were shown to be associated with periodontal disease (56). In our present study, OmpA was identified as the most abundant extracellular protein of *Aa*. This observation is consistent with the findings from previous studies (26, 56) and the demonstrated role of OmpA in serum resistance (57). Of note, OmpA is not only a major virulence factor in *Aa*, but it is also known to contribute to adhesion, biofilm formation, and immune activation in Acinetobacter baumannii and Escherichia coli (58). In our present study, OMPs were overall most abundantly detected in the media of *Aa* isolates with the serotypes b and c. Intriguingly, this implies that the extracellular abundance of OMPs is not directly related to the virulence of *Aa*. From a clinical perspective, the serotype a and b isolates are generally considered to be more virulent than serotype c isolates (59, 60). This view is mirrored in the results of our present virulence assays, where we show that the serotype c isolates display the lowest adherence to epithelial cells and, perhaps more importantly, the lowest cytotoxicity toward neutrophils and epithelial cells.

Interestingly, our *in vitro* infection assays showed a somewhat higher cytotoxicity of serotype a isolates toward salivary gland epithelial cells compared to serotype b isolates, and no clear differences in the virulence of serotype a and b isolates with respect to neutrophils. This contrasts to some extent with the results from other previous studies, where *Aa* isolates with serotype b were shown to display a higher *in vitro* virulence toward immune and nonimmune cells than *Aa* isolates with other serotypes (21, 61, 62). Furthermore, it has been reported that the virulence of *Aa* toward oral epithelial cells and macrophages may not follow serotype-specific patterns (63). It is presently difficult to reconcile these different observations, but they may relate to the usage of different isolates and strains, as well as different infection models and culture conditions. In particular, many previous studies used S strains, which have characteristics that differ substantially from the clinical R isolates (26). Furthermore, the presently investigated clinical R isolates were all obtained from patients with severe periodontitis, which may have resulted in a bias toward more virulent *Aa* isolates with serotypes a, b, and c. Nonetheless, although we observe serotype-specific virulence patterns, between the investigated *Aa* isolates with a particular serotype, we do observe differences in virulence. This is exemplified by the serotype a isolate 7R and the serotype b isolate 5R, which both display a very low cytotoxicity toward neutrophils.

Given the observed serotype and isolate-specific pathogenic potential, we sought to identify virulence factors with potential key roles in the different infection models by correlating their presence or abundance to the observed virulence of individual isolates. The results underscore the importance of fimbriae-related proteins in virulence, especially proteins encoded by the tight-adherence (*tad*) gene locus. This locus includes 14 genes (*flp-1*-*flp-2*-*tadV*-*rcpCAB*-*tadZABCDEFG*), which are responsible for adherence, autoaggregation, fimbrial expression, and pathogenesis in *Aa* (64, 65). In a rat infection model, a *tadA* mutant *Aa* strain failed to colonize and persist in the oral cavity, and it neither induced bone loss nor an IgG immune response (66). Furthermore, the TadD protein is known to be indispensable for the expression of fimbriae in *Aa*, and *tadD*-deficient bacteria displayed reduced auto-aggregation *in vitro* (67). Also, *rcpC* mutant *Aa* strains showed a reduced piliation (68).

Epithelial cells form the first barrier against colonization of the oral cavity by pathogens (69, 70). From the present data, we infer that the fimbriae-related TadA and RcpC proteins contribute to *Aa*’s ability to adhere to epithelial cells. Furthermore, the TadD and LolA proteins are associated with epithelial cell killing. An important role of the outer membrane lipoprotein chaperone LolA in the virulence of *Aa* is plausible because it has been shown for E. coli and Pseudomonas aeruginosa that LolA is important for cell growth (71, 72) and the virulence of P. aeruginosa (72). Also, LolA of Xanthomonas campestris *pv. campestris* has a role in the attachment to substrates and virulence (73). Considering the general role of LolA in lipoprotein sorting, it seems most likely that this chaperone has an indirect role in the virulence of *Aa*.

LtxA is a key extracellular virulence factor for *Aa*, which specifically induces the killing of human leukocytes. It is well known that the LtxA production varies among different *Aa* isolates. Particular serotype b isolates were previously shown to produce higher levels of LtxA than *Aa* isolates with other serotypes (40, 74). Consistent with this notion, our Western blotting results and exoproteome analyses show that the serotype b isolates generally secrete much more LtxA than the serotype a or c isolates that secreted comparable amounts of LtxA. The elevated production of extracellular LtxA may thus contribute to the relatively high pathogenicity of the serotype b isolates. On the other hand, the virulence of the serotype a isolates and especially their leukotoxicity is substantially higher than that of isolates with serotype c. This implies that, despite the previously proven role of LtxA in the virulence of *Aa*, other virulence factors like TadA, TadD, and OppA may have a higher contribution to the leukotoxicity of *Aa*. Here, it is noteworthy that leukotoxicity and NETosis are elicited to different extents by the investigated *Aa* isolates, as the leukotoxicity of serotype c isolates was marginal, whereas these isolates did elicit comparable levels of NETosis as the serotype a and b isolates. Especially the detection of TadD seems to be associated with NETosis. However, with respect to leukotoxicity, a possible limitation of the present study is that the physiochemical and nutritional conditions in BHI broth, used for our exoproteome analyses, are different from the intracellular conditions in our infection models. Consequently, it is hard to tell which virulence factors are exactly expressed and secreted once the *Aa* bacteria have been internalized by the neutrophils or epithelial cells. Another potential limitation in our study relates to the possibility that proteases released from LtxA-exposed neutrophils could potentially degrade LtxA (31).

CRISPR-Cas systems have been identified in many prokaryotes, where they play key roles in adaptive immunity (75). The known CRISPR-Cas systems can be divided into two classes, six types, and type I, II, and III systems. The type I system is the most widely distributed CRISPR-Cas system, but it can be subdivided into seven subtypes (I-A, I-B, I-C, I-D, I-E, I-F, and I-U) (76). Unexpectedly, we observed that the growth media of serotype a isolates of *Aa* contained type I Cas proteins. These proteins were neither identified for the serotype b or c isolates, nor the serotype a isolate 25R, which is due to the fact that these isolates lack the respective genes. Here, it must be noted that the 25R isolate is phylogenetically quite distinct from the other investigated serotype a isolates, which implies that the CRISPR-Cas systems are only present in some serotype a isolates of *Aa*. Importantly, it has been reported that type II CRISPR-Cas systems support the bacterial evasion from host defenses (77), and quite recently it was shown that a type I CRISPR-Cas system of P. aeruginosa has a role in immune evasion (78). It thus seems conceivable that the identified CRISPR-Cas systems may support immune evasion by the serotype a isolates of *Aa*, although this is not directly evident from our present investigation.

Lastly, for bacterial pathogens, the human body represents an iron-restricted environment and, accordingly, pathogens have evolved systems for “stealing” iron from their host. In our present study, the investigated serotype c isolates of *Aa* showed a lower virulence in our different infection models compared to the serotype a or b isolates. On the other hand, the serotype c isolates presented a higher abundance of the extracellular AfeA and FecB proteins associated with iron acquisition. These observations may suggest that the *Aa* isolates of serotype c can compensate their relatively lower virulence by a higher capacity for iron uptake. Conversely, this could imply that *Aa* isolates of serotypes a and b display an overall higher virulence to capture more iron from the host, which would for instance be more important from the bacterial point of view in anemic patients. Interestingly, it has been reported that the levels of hemoglobin, hematocrit values, and red blood cell counts can be correlated with chronic periodontitis (79). Also, after nonsurgical periodontal therapy, the anemic status of patients was reported to be improved and, according to a meta-analysis of 16 studies, periodontitis has the potential to disturb the balance in iron metabolism (80). While our present observations do not provide clues on how periodontal therapy could lead to an improved anemic status of patients in case of periodontitis caused by *Aa*, they do seem to suggest that the iron nutritional status of an individual may select for more or less virulent *Aa* isolates. In turn, this would mean that aggressive forms of periodontitis caused by *Aa*, especially in the case of serotype a and b isolates, could relate to the general nutritional status of the human host. If so, dietary iron supplementation would be a relatively simple approach to at least prevent *Aa*-inflicted aggressive forms of periodontitis.

## MATERIALS AND METHODS

### Bacterial isolates, cultivation, and monitoring of growth.

All 27 *Aa* isolates described in this study were collected from Dutch individuals during microbiological diagnosis of periodontitis. The serotypes of these isolates are summarized in Table 2. Based on medical-ethical considerations, metadata of the respective patients and their characteristics were not retained. The *Aa* isolates 4R, 5R, 23R, 30R, and 31R and the reference strain ATCC 29522 with serotype b were previously characterized (26). None of these serotype b isolates have the JP2 genotype and all were *cagE* positive (26). To identify representative *Aa* isolates with the serotypes a and c, our collection of *Aa* isolates from Dutch periodontitis patients was screened by PCR using serotype a-, c-, or f-specific primers (81). In brief, the primers used to identify serotype a isolates were 5′-TGGGTCATGGGAGGTACTCC-3′ and 5′-GCTAGGAACAAAGCAGCATC-3′, the primers to identify serotype c isolates were 5′-GAAACCACTTCTATTTCTCC-3′ and 5′-ARAAYTTYTCWTCGGGAATG-3′, and the primers to identify serotype f isolates were 5′-CCTTTATCAATCCAGACAGC-3′ and 5′-ARAAYTTYTCWTCGGGAATG-3′. PCR was performed using the following conditions: initial denaturation at 94°C for 5 min; amplification by 30 cycles at 94°C for 30 s, 55°C for 30 s, 72°C for 30 s; and final extension at 72°C for 5 min. The sequenced *Aa* isolates 14R (serotype a) and 1R (serotype c) were used as controls. The size of the serotype a-, c-, and f-specific amplified DNA fragments thus obtained was 293 bp, 268 bp, and 232 bp, respectively (see Fig. S1 in the supplemental material). The serotype a isolates 3R, 7R, 14R, 16R, and 25R, and the serotype c isolates 2R, 8R, 9R, 20R, and 27R were selected for further analyses. Furthermore, the *Aa* strain ATCC 29522, which belongs to the serotype b and displays an S colony phenotype was used as a reference strain.

**TABLE 2:** Serotypes of all clinical *Aa* study isolates

Serotype	Isolate name
a	3R, 7R, 14R, 15R, 20R, 25R
b	4R, 5R, 10R, 23R, 29R, 30R, 31R
c	1R, 2R, 6R, 8R, 9R, 11R, 16R, 17R, 18R, 24R, 27R
f	21R, 22R, 28R

Brain heart infusion (BHI) agar (Oxoid, UK) and broth (Oxoid, UK) supplemented with 5% l-cysteine, 5 mg/L hemin, and 1 mg/L menadione were used for *Aa* cultivation. To collect secreted proteins, the different *Aa* isolates were cultured for ~40 h in BHI broth at 5% CO_2_ and 37°C. At this time point, the bacteria had reached stationary phase as was determined by following the bacterial growth by counting CFU, as previously described (26). To ensure the homogeneity of cultures with *Aa* bacteria with the R phenotype, the culture media were always inoculated from frozen glycerol stocks that were kept at −80°C.

To determine the CFU of cultured *Aa* isolates with the R phenotype, the cultured bacteria were first dispersed by nine cycles of sonication with pulses of 5 s and intervals of 5 s, using a Misonix sonicator S-4000 (Amplitude 5). Subsequently, the dispersed bacteria were plated on BHI agar and CFU were counted upon incubation for 2 days. To establish calibration curves that correlate the optical density at 600 nm (OD_600_) of cultures of each R-type *Aa* isolate to CFU, the OD_600_ of different dilutions of the respective dispersed bacteria was measured and correlated with the respective CFU counts measured upon plating.

### Transmission electron microscopy.

The morphology of *Aa* was examined by TEM, as previously described (82). In brief, all *Aa* isolates were cultured on BHI agar plates for 40 h, as described above. The colonies were collected and washed twice with phosphate-buffered saline (PBS). Subsequently, the pelleted bacteria were fixed with 200 μL of 4% paraformaldehyde (PFA) before resuspension in 150 μL PBS. Aliquots of 5 μL of the bacterial suspension were placed on a copper grid, and the liquid was drained with Whatman filter paper. Subsequently, the bacteria on the grid were stained with 4% uranyl-acetic acid for 1 min with air drying. The samples were then inspected by TEM with a Talos F200i electron microscope at 80 Kv.

### Whole-genome sequencing and phylogenetic analysis.

Whole-genome sequencing of the selected *Aa* isolates was performed using the Nanopore MinION and Illumina Miseq platforms, as previously described (83). Subsequently, a hybrid assembly of the Nanopore and Illumina sequence data were performed using SPAdes version 3.11 (http://bioinf.spbau.ru/en) (83). Core genome single nucleotide polymorphisms (SNPs) were filtered based on hybrid assembly sequences of all *Aa* isolates using the CSI Phylogeny 1.4 algorithm provided by the Center for Genomic Epidemiology (http://www.genomicepidemiology.org/). Subsequently, the sequences of core SNPs of all *Aa* isolates were aligned to create a phylogenetic tree using Mega 11 (provided at https://www.megasoftware.net/) with the maximum likelihood method (84).

### Collection of extracellular proteins.

*Aa* isolates were cultured in BHI broth for 40 h and, subsequently, cells were separated from the growth medium by centrifugation. Extracellular proteins in the growth medium fraction were precipitated with trichloroacetic acid (TCA; Sigma-Aldrich, USA) at 4°C. The precipitated proteins were then collected by centrifugation (13,200 rpm), washed with pure acetone, dried at 60°C, and resuspended in 6 M urea, 0.1 M HEPES and 0.25 M NaCl. Subsequently, the concentration of extracellular proteins was measured with the Pierce bichinchoninic acid (BCA) protein assay kit (Thermofisher Scientific, USA), according to the manufacturer’s instructions. The extracellular proteins of each investigated *Aa* isolate were isolated from three independent replicate cultures.

### LDS-PAGE and Western blotting.

Samples of extracellular proteins (23 μg) were separated on 10% NuPAGE gels (Invitrogen, USA) by lithium dodecyl sulfate (LDS) polyacrylamide gel electrophoresis (PAGE). Upon electrophoresis, the separated proteins were stained with SimplyBlue SafeStain (Life Technologies, CA).

For Western blotting analyses of LtxA production, the separated proteins were transferred from the gel to a Protran nitrocellulose transfer membrane (Whatman, Germany) by semidry blotting. Subsequently, the membrane was incubated with the LtxA-specific monoclonal antibody 83 (30). LtxA-specific protein bands were visualized with an IRDye 800CW-labeled secondary goat-anti mouse antibody and scanning of the membrane with an Amersham Typhoon biomolecular imager (Cytiva, Medemblik, the Netherlands).

### Sample preparation for mass spectrometry and data processing.

Extracellular proteins were prepared for MS/MS analyses as previously described (85). In brief, for each *Aa* isolate, a total amount of 37.5 μg of extracellular proteins was separated on 10% NuPAGE gels with a separation distance of 1 cm. Subsequently, the gel was stained with SimplyBlue SafeStain. The different lanes with separated proteins were excised from the gel and then sliced into smaller pieces that were transferred to low-binding tubes. Once the gel pieces were destained and dried in a vacuum centrifuge, a solution of 0.02 μg/mL trypsin (Promega, USA) was added, and the proteins were digested overnight at 37°C. The resulting tryptic peptides were eluted in water using an ultrasonic bath. The supernatant containing peptides was then transferred into a fresh vial and concentrated in a vacuum centrifuge. Lastly, the dried peptides were resuspended in 0.1% (vol/vol) acetic acid.

LC-MS/MS analyses were performed with an EASY-nLC II liquid chromatography system coupled to a LTQ Orbitrap (ThermoFisher Scientific, USA). In particular, the tryptic peptides were loaded on a self-packed analytical column (OD 360 μm, length 20 cm) filled with 3-μm diameter C18 particles (Maisch HPLC GmbH). Peptides were then eluted using a binary nonlinear gradient of 5 to 99% acetonitrile in 0.1% acetic acid over 151 min at a flow rate of 300 nl/min, and subjected to electrospray ionization-based MS. A full scan in the Orbitrap with a resolution of 30,000 was followed by collision-induced dissociation (CID) of the five most abundant precursor ions. MS/MS experiments were acquired in the linear ion trap.

A nonredundant database for protein identifications was created by RAST annotation of the hybrid-assembled whole-genome sequences of the 15 selected *Aa* isolates with serotypes a, b, or c. Importantly, the resulting nonredundant database with 11,156 protein sequences and Uniprot identifiers also included homologous proteins with only one amino acid difference. Subsequently, the database was supplemented with protein sequences of Bos taurus, retrieved from Uniprot on 5 December 2019 (Proteome ID 9913), as well as common laboratory contaminants and a reverse entry for every forward entry, resulting in a total of 115,316 entries. Database searching was then performed with Sorcerer-SEQUEST 4 (Sage-N Research, USA) by applying fully specific tryptic cleavage (KR/P) with up to two missed cleavages and methionine oxidation (+15.99 Da) as variable modifications. The precursor mass tolerance was set to 10 ppm and the fragment mass tolerance was set to 0.5 Da. Validation of the MS/MS-based peptide and protein identifications was performed using Scaffold (version 4.8.7; Proteome Software), and peptide identifications were accepted if they at least exhibited deltaCn scores of greater than 0.1 and XCorr scores of greater than 2.2, 3.3, and 3.75 for doubly, triply, and all higher charged peptides, respectively. Lastly, protein identifications were accepted if at least two unique peptides were identified in two out of three replicates with the above criteria. With these filter parameters, <0.5% false-positive hits were obtained. For protein quantification, NSAF values (86) were exported. All data were exported from Scaffold to Microsoft Excel for further investigation. The NCBI identifier was used to enhance the annotation of identified proteins as presented in Table S1.

### Cell lines culture conditions.

**(i) A253 epithelial cells.** Salivary gland A253 epithelial cells were cultured in Dulbecco modified Eagle medium (DMEM)-GlutaMAX medium (Thermo Fisher Scientific, USA) supplemented with 10% fetal calf serum (Sigma-Aldrich, USA) at 37°C, 5% CO_2_.

**(ii) Human neutrophils.** Blood donations were received from healthy volunteers, who had been medically examined. Fresh neutrophils were isolated using Lymphoprep buffer (Stemcell, USA) as previously described (87). The isolated neutrophils were resuspended in Roswell Park Memorial Institute 1640 (RPMI) medium (Thermo Fisher Scientific, USA) supplemented with 2 mM l-glutamine and 10% donor plasma at 37°C and 5% CO_2_. To perform neutrophil extracellular traps (NETs) formation assays, the neutrophils were incubated in RPMI medium supplemented with 2% or 10% fetal calf serum.

### Assessment of *Aa* interactions with epithelial cells and neutrophils.

**(i) *Aa* interaction with epithelial cells.** Epithelial cells were seeded at a density of 1.5×10^5^/well in 24-well plates and incubated for 2 days. Prior to infection, the wells were washed with PBS to remove nonadherent epithelial cells and, subsequently, the epithelial cells were challenged with *Aa* at a multiplicity of infection (MOI) of 100 for 2 h. After infection, the epithelial cells were washed five times with PBS and lysed with 0.1% Triton-X in PBS for 5 min at 37°C and 5% CO_2_. The diluted cell lysates were then plated on BHI agar and incubated for 3 days. The number of bacteria released from the lysed cells was determined by CFU counting.

**(ii) *Aa* cytotoxicity assays.** To measure the ability of *Aa* to kill epithelial cells and neutrophils, lactate dehydrogenase (LDH) release assays (88) were performed with the LDH cytotoxicity assay kit (Thermo Fisher Scientific, USA) according to the manufacturer’s instructions. Briefly, a total number of 3.5×10^4^ epithelial cells or neutrophils was incubated per well of 96-well tissue culture plates. The epithelial cells (in DMEM) and neutrophils (in RPMI) were challenged with *Aa* isolates at an MOI of 100 for 6 h or 2 h, respectively, as previously described (26). To assess the maximal LDH activity, the epithelial cells or neutrophils were incubated in the lysis buffer provided with the LDH assay kit. As a control for spontaneous LDH release, epithelial cells and neutrophils were incubated with PBS or MilliQ water, respectively. To quantify the LDH release, the absorbance of the suspensions was measured at 490 nm, using a subsequent read-out at 680 nm to determine the background signal. The cytotoxicity was then calculated using the following formula: percentage cytotoxicity = (*Aa*-treated LDH activity − spontaneous LDH activity/maximum LDH activity − spontaneous LDH activity) × 100.

**(iii) NETs formation.** NETs formation was assessed as previously described with modifications (89). Briefly, 3.75×10^4^ neutrophils were seeded per well of a black flat bottom well plate (Thermo Fisher Scientific, USA). The neutrophils were challenged with *Aa* at an MOI of 100 for 2 h before 2 μM the ‘red fluorescent cell linker’ (Sigma-Aldrich, USA) was added to stain the cells. As a positive control for NETosis, the neutrophils were incubated with 20 nM phorbol 12-myristate 13-acetate (PMA), whereas fresh RPMI culture medium was added as a negative control. To stain extracellular DNA, the neutrophils were incubated with 5 μM the membrane-impermeable DNA dye Sytox green (Thermo Fisher Scientific, USA) for 15 min. After fixation in 100 μL with 4% PFA, NETs were visualized by confocal immunofluorescence microscopy (Zeiss Celldiscoverer 7).

All experiments were performed as three biological replicates with triplicate measurements per condition.

### Data analysis.

SignalP (version 5.0) (90), SecretomeP (version 2.0) (91), TMHMM (version 2.0) (92), and LipoP (version 1.0) (92) were used to predict signal sequences, transmembrane α-helixes and proteins with a lipobox, respectively. To predict the extracellular protein localization, UniProt, PsortB (version 3.0.3) (93), ProtComP (version 9.0), and CELL-PLoc (version 2.0) (94) were used. A principal-component analysis (PCA) was performed to evaluate the relationship between different serotypes with ClustVis (95). For visualization of protein functions, Voronoi treemaps were built based on gene ontology (GO) terms using Paver (version 2.1) (Decodon GmbH, Greifswald, Germany) (96). Of note, protein abundances measured as NSAF values were considered to be significantly different when the respective *P* value was lower than 0.01 in a Student's *t* test and the log_2_ fold change value was higher than 0.8. If a particular protein was identified in the exoproteomes of *Aa* isolates belonging to one serotype, but not in any triplicate data sets from another serotype, an artificial number of 100 or −100 was attributed to this protein. Virulence genes were predicted with the VirulentPred algorithm, which predicts bacterial virulence factors based on their amino acid composition, the Cascased bilayer cascade Support Vector Machine module (SVM) (97), and the Predict Pathogenic Proteins in Metagenomic data sets (MP3) website (98). Furthermore, subcellular localization predictions and virulence gene predictions were evaluated by manual curation based on published literature data.

Statistical analyses were performed with GraphPad Prism 8.0.1. Data sets from cytotoxicity studies with mammalian cells relating to three or more unmatched groups were statistically evaluated with Kruskal-Wallis tests and subsequent Dunn’s or Dunnett’s multiple-comparison tests. Statistical differences in CFU assays were assessed using two-way ANOVA tests and subsequently by the Šidák multiple-comparison test. A *P* value of < 0.05 was considered statistically significant.

### Biological and chemical safety.

*Aa* was handled following appropriate safety procedures for biosafety level 2 (BSL-2) microbiological agents. All chemicals and reagents used in this study were handled according to the local guidelines for safe handling and protection of the environment.

### Ethics statement.

Blood donations from healthy human volunteers were collected based on written informed consent with approval of the medical ethics committee of the University Medical Center Groningen (UMCG; approval no. Metc2012-375), and in accordance with the Declaration of Helsinki guidelines.

### Data availability.

The results of whole-genome sequences were submitted to GenBank (NCBI) through BioProject number PRJNA787784 under accession numbers JALDMS000000000 (isolate 2R), JALDMT000000000 (isolate 3R), JALDMU000000000 (isolate 7R), JALDMV000000000 (isolate 8R), CP093913 (isolate 9R), CP093912 (isolate 14R), CP093911 (isolate 16R), JALDMW000000000 (isolate 20R), JALDMX000000000 (isolate 25R), and CP093910 (isolate 27R). Genome sequences of the serotype b isolates were previously published and submitted to NCBI (26).

The results of proteome analyses were submitted to the ProteomeXchange Consortium via the PRIDE partner repository with the data set identifier PXD036160.
