# Supplementary material for: Differential Virulence of Aggregatibacter actinomycetemcomitans Serotypes Explained by Exoproteome Heterogeneity
Source: Microbiol Spectr. 2022 Dec 21;11(1):e03298-22. doi: 10.1128/spectrum.03298-22 (PMC9927298; doi:10.1128/spectrum.03298-22)
Supplement: Supplemental file 1 — Fig. S1 to S3. Download spectrum.03298-22-s0001.pdf, PDF file, 5.0 MB [file spectrum.03298-22-s0001.pdf]

Supporting information

Differential virulence of *Aggregatibacter actinomycetemcomitans* serotypes explained by exoproteome heterogeneity

Yanyan Fu[a], Sandra Maaβ[b], Francis M. Cavallo[a], Anne de Jong[c], Erwin Raangs[a], Johanna Westra[d], Girbe Buist[a], Dörte Becher[b], and Jan Maarten van Dijl[a]

[a]University of Groningen, University Medical Center Groningen, Department of Medical Microbiology, Groningen, the Netherlands

[b]University of Greifswald, Institute of Microbiology, Department of Microbial Proteomics, Greifswald, Germany

[c]University of Groningen, Groningen Biomolecular Sciences and Biotechnology Institute, Department of Molecular Genetics, 9747 AG Groningen, the Netherlands

[d]University of Groningen, University Medical Center Groningen, Department of Rheumatology and Clinical Immunology, Groningen, The Netherlands

# Correspondence: Jan Maarten van Dijl, University Medical Center Groningen, Department of Medical Microbiology, Hanzeplein 1, 9700RB Groningen, the Netherlands, tel. +31-50-3615187, e-mail: j.m.van.dijl01@umcg.nl

Running title: Virulence of *Aggregatibacter actinomycetemcomitans* in different serotypes

Table of Contents:

Supplementary Figure S1. Selection of representative *Aa* isolates with serotype a or c.

Supplementary Figure S2. Principal component analysis (PCA) of all identified extracellular proteins of serotype a, b and c except the outer membrane proteins (OMPs).

Supplementary Figure S3. Voronoi treemaps of extracellular proteins in *Aa* isolates with differernt serotypes.

Available separately:

Supplementary Table S1. All extracellular proteins of *Aa* bacteria with serotype a, b and c identified by Mass Spectrometry and their respective normalized spectral counts values..

Supplementary Table S2. Analysis of the statistical significance of differences in the virulence of *Aa* in several different human cell models.

Supplementary Table S3. Correlation between identified virulence factors of *Aa* and virulence in different cell infection models.

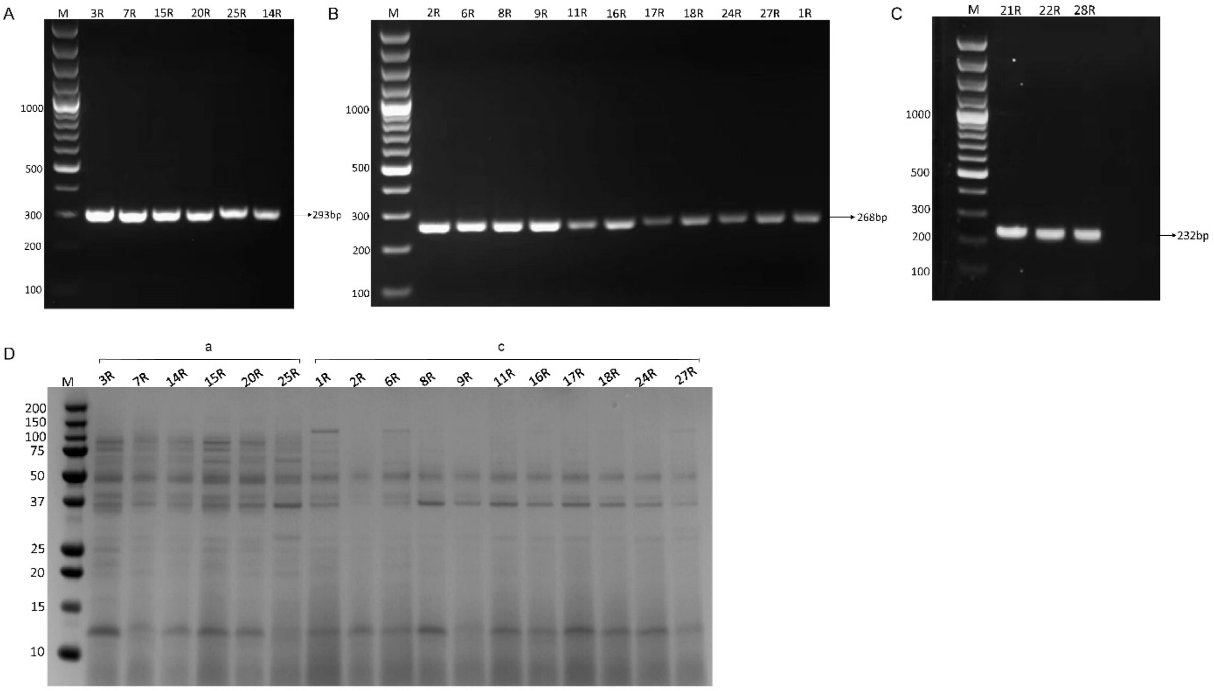

Supplementary Figure S1. Selection of representative *Aa* isolates with serotype a or c. PCR was performed with *Aa* serotype-specific primers as specified in the Materials and Methods section. Next the PCR products obtained from the identified serotype a (A), serotype c (B), and serotype f (C) isolates were separated on 1% agarose gel. (D) LDS-PAGE analysis of extracellular proteins of *Aa* with serotype a and c. The gel was stained by SimplyBlue staining. The Molecular weight of marker proteins is indicated on the left.

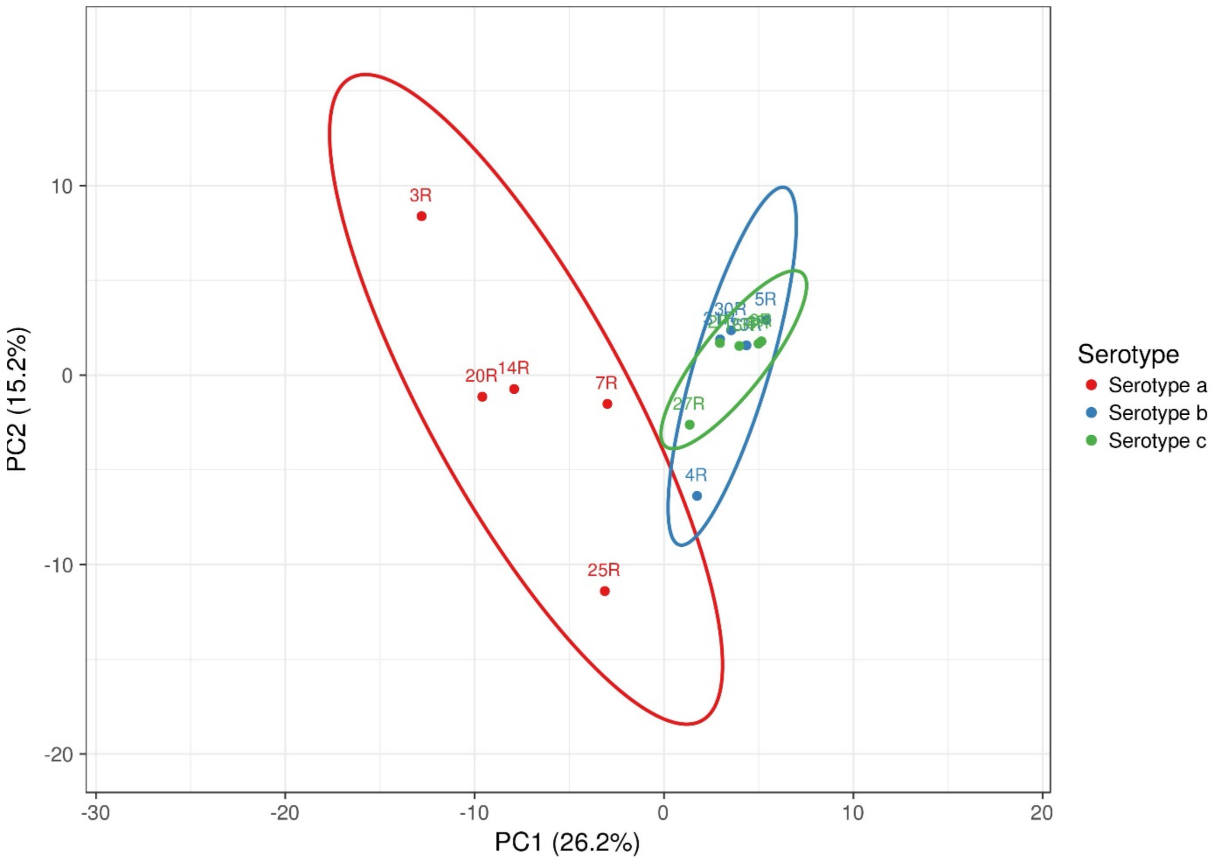

Supplementary Figure S2. Principal component analysis (PCA) of all identified extracellular proteins of *Aa* with serotypes a, b or c except the outer membrane proteins (OMPs). The plot is based on the normalized spectral counts of all identified proteins of these three serotypes except OMPs.

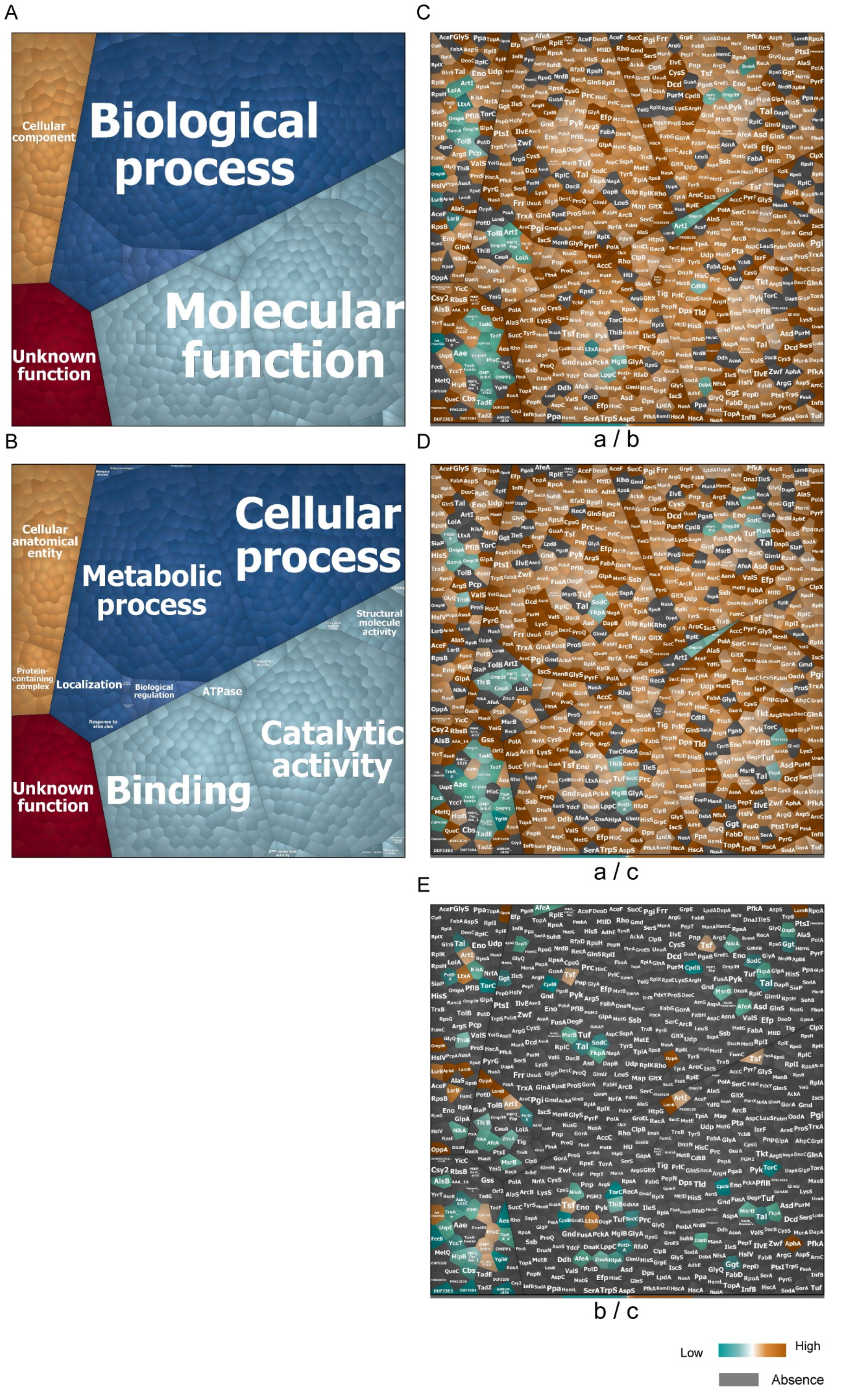
A
Cellular component
Biological process
Molecular function
Unknown function
B
Cellular anatomical entity
Metabolic process
Cellular process
Structural molecule activity
Protein-containing complex
Localization
Biological regulation
ATPase
Response to stimulus
Catalytic activity
Binding
Unknown function
C
a / b
D
a / c
E
b / c
Low
High
Absence

Supplementary Figure S3. Voronoi treemaps of extracellular proteins from all *Aa* isolates with different serotypes. The functions of all identified extracellular proteins were predicted based on GO terms. The above panels show the functional categories of all identified extracellular proteins. The top-level functional categories of all identified extracellular proteins are indicated in (A), and the second-level functional categories are represented in (B). The panels C-E show the names of the different identified proteins within the respective functional categories with pairwise comparisons: (C) shows the comparison for the serotypes a versus b, (D) for serotypes a versus c, and (E) for serotypes b versus c. The images correspond to Figure 7 in the main manuscript.
